# A major ecological niche of eosinophils in evolving *Schistosoma* granulomas challenges the eosinophil view as “helminth killer” cells

**DOI:** 10.1126/sciadv.adt2779

**Published:** 2025-06-11

**Authors:** Luccas M. Barata, Kássia K. Malta, Vitor H. Neves, Cinthia Palazzi, Eliane G. Oliveira-Barros, Yasmin Aguiar, Felipe Kneip, Bruno A. Marcelino, Lívia A. S. Carmo, João Felipe Audi-Gazeta, Pedro H. S. Santos, Maria Karolynna B. Milani, Michelle C. A. Paula, Eliana C. B. Toscano, Rosana Gentile, Felipe F. Dias, Thiago P. Silva, Rossana C. N. Melo

**Affiliations:** ^1^ Laboratory of Cellular Biology, Department of Biology, Institute of Biological Sciences, Federal University of Juiz de Fora (UFJF), Rua José Lourenço Kelmer, Juiz de Fora, MG 36036-900, Brazil.; ^2^ Cell Biology Graduate Program, Federal University of Minas Gerais (UFMG), Belo Horizonte, MG, Brazil.; ^3^ Biodiversity and Nature Conservation Graduate Program, UFJF, Juiz de Fora, Brazil.; ^4^ Laboratory of Communicable Diseases, Department of Parasitology, Aggeu Magalhães Institute, Oswaldo Cruz Foundation, Recife, PE, Brazil.; ^5^ Laboratory of Pathology, Department of Pathology, Federal University of Juiz de Fora Medical School, Juiz de Fora, MG, Brazil.; ^6^ Laboratory of Biology and Parasitology of Wild Reservoir Mammals, Oswaldo Cruz Foundation, Rio de Janeiro, Brazil.; ^7^ Laboratory of Cellular Biology, Department of Biological Sciences, State University of Minas Gerais (UEMG), Campus Ibirité, Ibirité, MG, Brazil.

## Abstract

Eosinophil-rich granulomas, formed around tissue-trapped parasite eggs, are hallmarks of schistosomiasis mansoni, a prevalent neglected tropical disease. How eosinophils populate and affect the complex *Schistosoma* granulomas remains unclear. Here, we mapped eosinophils across evolutional hepatic granulomas in a mouse model and in a primary wild reservoir for human schistosomiasis in Brazil (water rat *Nectomys squamipes*). With in-depth quantitative image analysis and three-dimensional histological reconstructions of entire granulomas, we find that eosinophils are spatially organized and occupy a major, peripheral niche conserved across space and time in all granuloma stages and both experimental and natural infections. Within this niche, immature and mature eosinophils coinhabit, compartmentalize their major basic protein-1 content, robustly interact with other immune cells, and secrete through piecemeal degranulation. This unveiled niche, unrelated to parasite eggs, challenges the concept of eosinophil as a “helminth killer” cell and invigorates its view as an immunoregulatory cell of the tissue microenvironment in *Schistosoma* granulomas.

## Teaser

Discoveries shift understanding of eosinophils in schistosomiasis mansoni.

## INTRODUCTION

Schistosomiasis, an ancient disease caused by trematode worms of the genus *Schistosoma*, continues to plague millions across the globe with its parasitic embrace (*1*, *2*). From a global public health perspective, schistosomiasis is considered the most important water-based disease, still spreading into nonendemic areas in parallel with human migratory flows and management of water resources in tropical and subtropical climate zones (*3*, *4*).

Schistosomiasis has been reported from 78 countries and is considered an “emblem of how hard it is to prevent, control, and treat a neglected tropical disease” (*3*). To date, no effective vaccine against this disease is available and treatment relies on a single drug—the anthelmintic praziquantel—with incomplete efficacy, limited supply and accessibility, and possibility of resistance (*5*). Schistosomiasis is also an emblematic example of how challenging it is to decipher the inner workings of a parasitic disease. Despite major advances in understanding this disease, the most prominent pathological feature of schistosomiasis—the granuloma—is still unresolved. *Schistosoma* granulomas are classically defined as highly organized immune cell aggregates settled around the parasite eggs trapped in target organs, mainly liver and intestines (*6*, *7*). Granulomas manifest prominently during schistosomiasis and have been paradoxically attributed with dual functions: serving as protective mechanisms for the host and conferring benefits to the parasite (*6*).

Our group has been rethinking the *Schistosoma* granuloma as an integrating and evolving ecosystem in which distinct cell populations colonize these structures in different space-time frames and establish complex interactions (*8*). In particular, substantial attention has been directed toward the population of eosinophils, cells of the innate immune system, due to their notable accumulation within *Schistosoma* granulomas (*8*–*10*).

Historically, eosinophils have been related to host protection against helminths like *Schistosoma mansoni*, acting as destructive effector cells. This concept was substantiated by a sequence of works, mostly conducted during the 1970s, demonstrating eosinophil-mediated killing of *S. mansoni* larvae (schistosomula) and eggs in vitro (*11*–*20*). The idea that eosinophils are attracted, firmly adhere to *S. mansoni* surface, an interaction mediated by a ligand (IgG or C3 complement) (*21*), and destroy this parasite through the release of their toxic granule-derived cationic proteins (*20*, *22*) strongly supported eosinophils as a “helminth killer” cell. However, the understanding of eosinophils as effector cells directly targeting the parasite lacks empirical validation from in vivo studies and the role of eosinophils during schistosomiasis remains speculative or controversial (*9*, *10*).

A major challenge in comprehending the core function(s) of eosinophils in schistosomiasis is the intricacies of eosinophil-rich granulomas induced by this infection. As evolutionary primitive structures, *Schistosoma* granulomas modify their microarchitecture and cell community over time (*8*, *10*). Although the formation of evolving granulomas is long recognized as a feature of the immune response to both human and experimental schistosomiasis mansoni (*23*), this fundamental evolutive aspect has garnered limited attention within the scope of research on the eosinophil biology associated with this helminthiasis. Therefore, it is essential to explore eosinophils within the granuloma framework.

In this study, we have investigated the spatial localization of eosinophils in numerous evolving granulomas formed in the liver during schistosomiasis mansoni. We provide in-depth quantitative image analysis and three-dimensional (3D) histological reconstructions of entire granulomas using two models of *S. mansoni* infection: an experimental (mice) and a natural (the neotropical, wild reservoir for human schistosomiasis *Nectomys squamipes*). We identified that, during the entire granuloma life, most eosinophils occupy a major, peripheral ecological niche in which these cells establish tight interactions with other immune cells. This uncovered spatial pattern, conserved in evolutional granulomas from both experimental and natural infections, and unassociated with parasite eggs, robustly supports a scenario inconsistent with a direct role for eosinophils toward the parasite.

## RESULTS

### The *Schistosoma* granuloma is a dynamic evolutionary system

To better characterize the process of granuloma formation taking place in hepatic schistosomiasis, we microscopically examined a large tissue area (total of 1368 mm^2^) of mice (*n* = 6 per group) experimentally infected with *S. mansoni*, a well-established animal model for this disease (*24*, *25*). Samples were taken on day 55 postinfection, corresponding to a fully developed acute phase characterized by an intense release of soluble egg antigens (SEAs), which trigger a robust T helper 2 (T_H_2) response, and on day 120 postinfection, corresponding to the chronic phase, marked by continuous egg deposition (*24*–*26*).

**Fig. 1.**
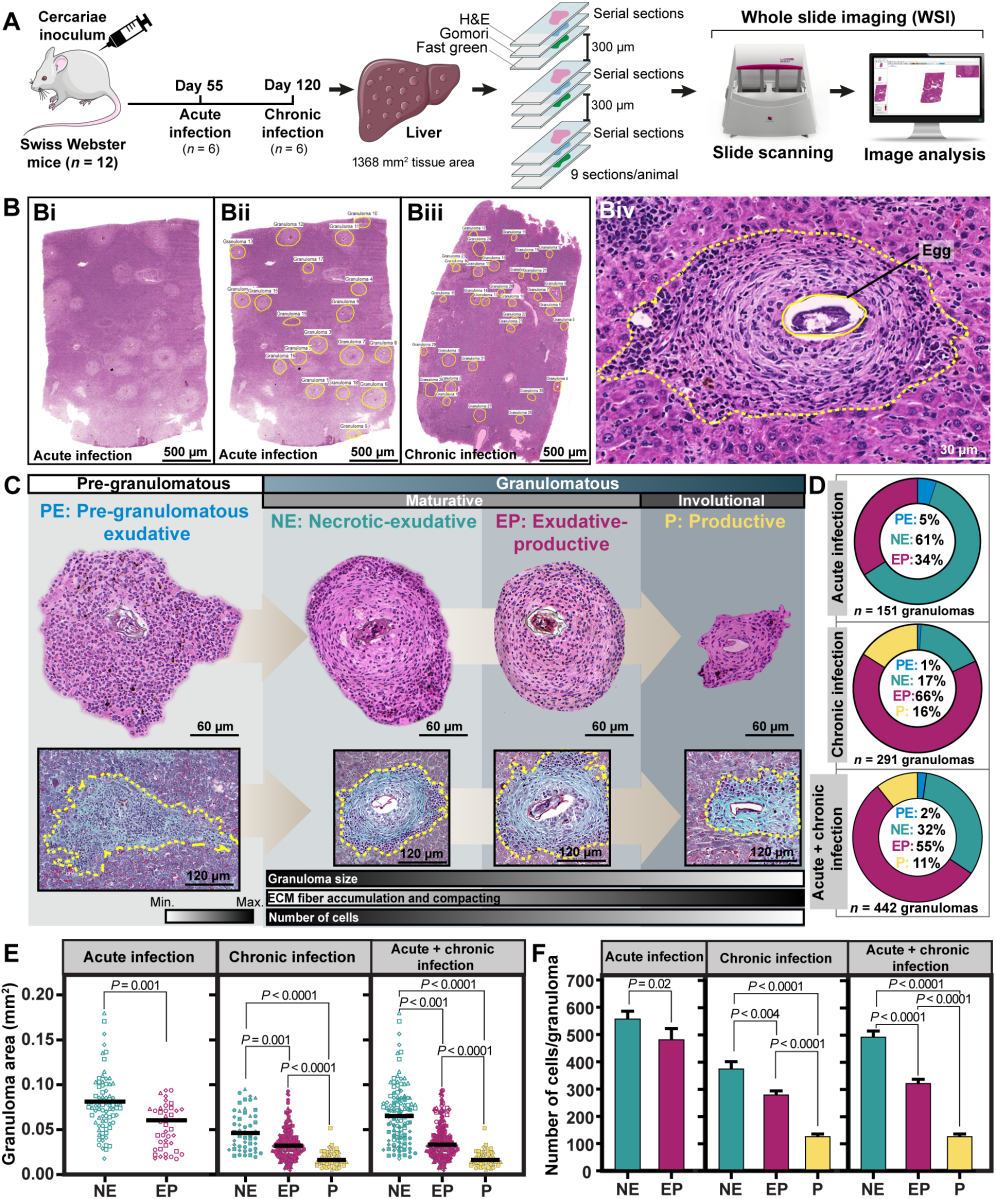
The *Schistosoma* granuloma is a dynamic evolutionary system. (**A**) Experimental setup to analyze the histopathology of hepatic granulomas in mice with acute and chronic *S. mansoni* infection. Animals were infected with a single inoculum of 100 cercariae, LE strain. Nine hepatic sections were obtained per animal. Each group of three sections was serially acquired, stained with three different stains (H&E, Gomori’s trichrome, and Fast green–Neutral red), and digitalized through WSI. A 300-μm interval was maintained between groups of three sections to deepen the organ and avoid reanalysis of the same granuloma. (**B**) In WSI scans of H&E-stained sections (**Bi**), all granulomas (total *n* = 442; 151 from acute phase and 291 from chronic phase) were outlined, enumerated, and classified per evolutive stage (**Bii** to **Biv**). (**C** and **D**) Pre-granulomatous and granulomatous phases and their evolutive stages (types and proportions). Granulomas were stained with H&E and Gomori’s, the latter of which stains ECM fibers, particularly collagen fibers, in green (*n* = 6 animals per group). Progressive accumulation of collagen fibers was observed. (**E** and **F**) The granuloma size and its total number of cells were significantly reduced alongside granuloma life. Granuloma area (mm^2^) was determined for individual granulomas. Scattered symbols in (E) represent single granuloma data. Different symbols represent individual animals [acute infection (*n* = 4), chronic infection (*n* = 4), and acute + chronic infection (*n* = 8)]. A total of 142,246 cells (71,964 from 151 acute granulomas and 70,282 from chronic granulomas) were quantified and the mean number of cells was established per granuloma type. Results are expressed as means ± SEM. *P* as indicated by the Mann-Whitney test (acute infection) or Kruskal-Wallis test followed by Dunn’s multiple comparisons test (chronic and acute + chronic infection).

To study liver histopathology in greater detail, we used a histological approach that integrates optimal fixation and processing with a plastic resin [glycol methacrylate (GMA)] embedding (*27*). This method offers enhanced tissue resolution and improved visualization of inflammatory processes compared to traditional paraffin embedding (*25*, *27*). In total, nine sections were collected per animal. Each group of three sections was serially acquired and stained with three different stains [hematoxylin and eosin (H&E); Gomori’s trichrome, and Fast green–Neutral red] to facilitate the identification of tissue and cellular morphological aspects (Fig. 1A). Then, a 300-μm interval was maintained between groups of three sections to deepen the organ and avoid reanalysis of the same granuloma (*25*). All sections were analyzed through whole slide imaging (WSI) (Fig. 1A).

WSI enables digitalization of entire sections with the use of a digital slide scanner, thus generating high-resolution and wide-field microscopy images with detailed information about the tissue microarchitecture (*28*) (Fig. 1A). WSI revealed marked formation and extensive distribution of granulomas in the entire hepatic parenchyma in both acute and chronic infections (Fig. 1B and fig. S1). First, to quantitate granulomas, H&E-stained WSI sections were analyzed and all granulomas (total *n* = 442 granulomas; 151 from acute and 291 from chronic infections) were outlined (Fig. 1, Bi to Biv).

Next, on the basis of histopathological features (*23*, *24*), all granulomas were classified per their evolutive stage (Fig. 1C) as detailed in fig. S1. As reported before (*23*, *24*), the process of granuloma formation involved a pre-granulomatous and a granulomatous phase. The pre-granulomatous phase [termed pregranulomatous-exudative (PE) stage] is characterized by a disordered collection of cells around the parasite egg, which progresses to a highly organized assembly of cells and extracellular matrix (ECM) fibers (granulomatous phase) (Fig. 1C and fig. S1). Once established, *Schistosoma* granulomas tend to be spherical and more compact, evolving from a maturative to an involutional state (Fig. 1C and fig. S1). Thus, recapitulating the human disease, our murine model of *S. mansoni* infection developed multiple granulomas seen at distinct evolutional stages side by side in the same tissue section (Fig. 1C and fig. S1).

As a general feature of both acute and chronic infections, the initial stage (PE) was found in lesser proportion compared to the other stages (Fig. 1D). The two mature forms of granulomas [termed necrotic-exudative (NE) and exudative-productive (EP)] (Fig. 1C and fig. S1) were the most frequent types of granulomas found in the hepatic parenchyma in both infection phases (Fig. 1D), whereas the involutive form [termed productive (P)] (Fig. 1C and fig. S1) was observed only in the chronic infection (Fig. 1D). Thus, during its life, the *Schistosoma* granuloma gradually undergoes involution, reducing in size (area) with the disease progression (Fig. 1, C to E).

Next, we evaluated the total number of cells and deposition of ECM fibers within granulomas. Our quantitative analyses of 142,246 cells (71,964 from 151 acute granulomas and 70,282 from 291 chronic granulomas) showed that the total number of cells progressively reduces alongside granuloma maturation and involution (Fig. 1F and table S1). In parallel, and confirming the pro-fibrotic property of *Schistosoma* granulomas (*6*, *23*), there was increased accumulation and compacting of ECM fibers, mainly collagen fibers, which indicates the establishment of fibrosis, as observed in Gomori-stained sections (Fig. 1C and fig. S2).

In line with our previous proposed viewpoint of a *Schistosoma* granuloma as an evolving ecosystem (*8*), we thus confirmed that the fundamental nature of the *Schistosoma* granuloma relies on an evolutionary perspective gradually encompassing changes in its microstructure.

### Eosinophils populate a preferential site within *Schistosoma* granulomas

**Fig. 2.**
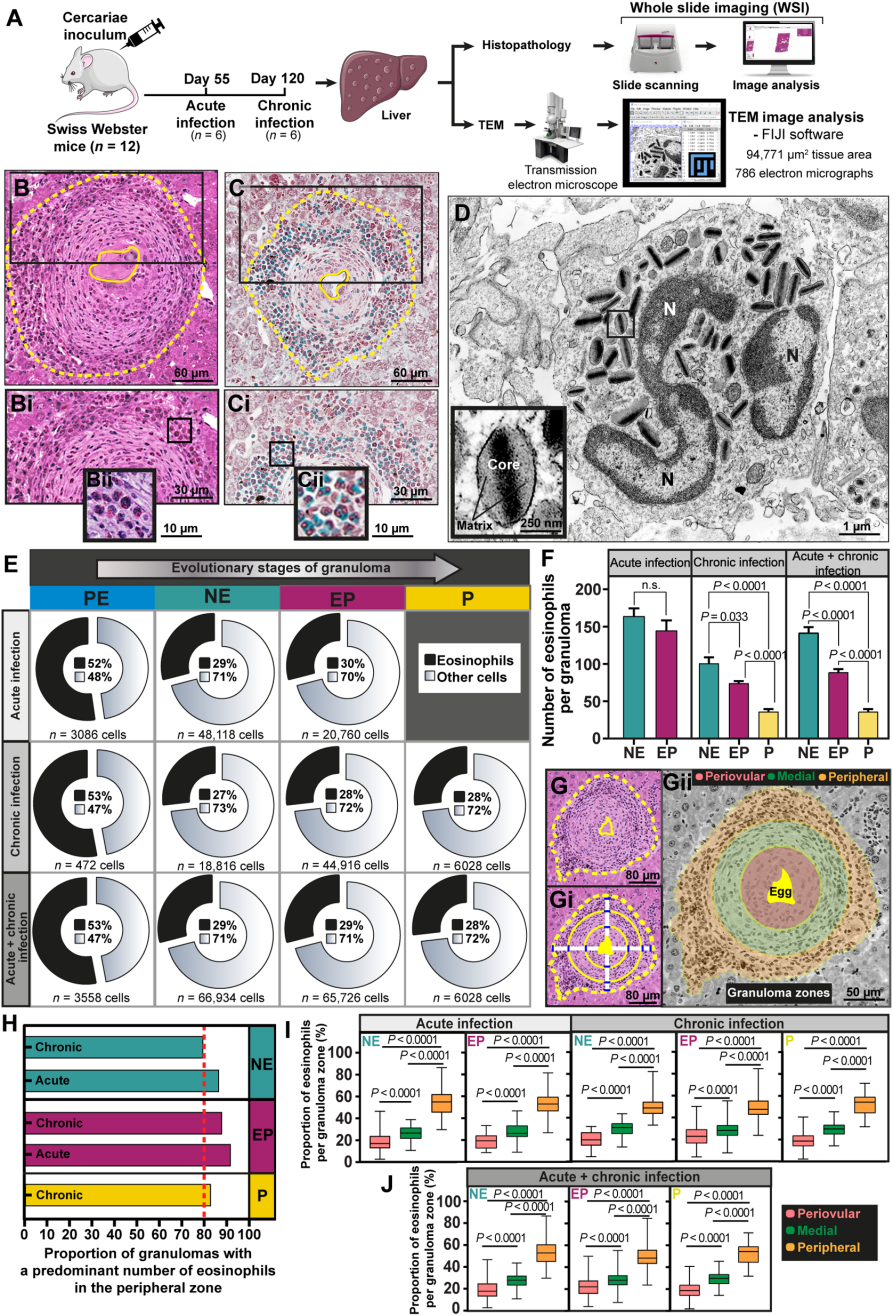
Infiltrating eosinophils are spatially organized and populate a preferential site within granulomas. (**A**) Experimental setup to investigate eosinophil numbers and distribution within hepatic granulomas in mice with acute and chronic *S. mansoni* infection. Eosinophils were evaluated by both WSI of histological sections (*n* = 3 sections per animal for each staining) and TEM. (**B** and **C**) Representative granulomas showing eosinophils organized in layers and identified by their segmented nuclei and acidophilic cytoplasm stained in pink (H&E) or green (Fast green–Neutral red), respectively. The boxed areas in (B) and (C) are shown at higher magnification in (**Bi** and **Bii**) and (**Ci** and **Cii**). (**D**) A representative electron micrograph depicts the unique eosinophil ultrastructure. Secretory granules (observed at higher magnification in the boxed area) display a central electron-dense core and outer electron-lucent matrix. N, nucleus. (**E**) Proportions of eosinophils within evolutional granulomas. The total number of cells/granuloma type is indicated. (**F**) The number of eosinophils/granuloma type significantly decreased alongside granuloma maturation and involution. Results are expressed as means ± SEM. *P* as indicated by the Mann-Whitney test (acute infection) and Kruskal-Wallis test followed by Dunn’s multiple comparisons test (chronic and acute + chronic infection). n.s., not significant. (**G**, **Gi**, and **Gii**) Schematic representation of three spatial zones (peripheral, medial, and periovular) in a representative granuloma. (**H**) At least 80% of all granulomas (total *n* = 424) exhibited most eosinophils in their peripheral zone. (**I** and **J**) Eosinophil numbers were significantly higher in the granuloma peripheral zone compared to the medial and periovular zones. Results are expressed as means ± SEM. *P* as indicated by one-way ANOVA followed by Tukey’s multiple comparisons test [acute infection (NE and EP), chronic infection (NE and P)] or Kruskal-Wallis test followed by Dunn’s multiple comparisons test [chronic infection (EP) and acute + chronic infection (NE and EP)].

The parasite eggs trapped in the liver attract a substantial population of eosinophils, which colonize the hepatic tissue to form granulomas together with other immune and resident cells (*29*). Infiltrating eosinophils were evaluated by both WSI of histological sections and transmission electron microscopy (TEM) (Fig. 2A). In WSI scans of liver sections, eosinophils within granulomas were unambiguously identified by their segmented nuclei and typical highly acidophilic nature of their cytoplasmic secretory granules (specific granules), which appear pink (Fig. 2, B, Bi, and Bii) or green (Fig. 2, C, Ci, and Cii), when stained with H&E or Fast green–Neutral red, respectively. The accumulating presence of eosinophils was also confirmed with TEM, which revealed the unique ultrastructure of eosinophil granules with a crystalline electron-dense core and an outer, electron-lucent matrix (*30*) (Fig. 2D). A total of 45,125 eosinophils within 442 granulomas (table S1) were quantified and the proportion of these cells per granuloma stage was determined compared to the total number of cells (total *n* = 142,246 cells) (Fig. 2E).

The pre-granulomatous (PE) stage, i.e., tissue sites in which immune cells are accumulating and in the process of organization into granulomas (Fig. 1C and fig. S1) depicted more than 50% of their total number of cells composed only of eosinophils (Fig. 2E). This initial accumulation of eosinophils may be important to orchestrate the immune response by attracting other cells to the site of infection. However, following granuloma formation in both acute and chronic infections, the proportion of eosinophils was smaller compared to the PE stage and remained stable in relation to the whole cell community (range of 27 to 30% of all granuloma cells) in all types of granulomas (Fig. 2E). Quantitative analyses showed that, alongside the decrease of the total number of cells within the granuloma (Fig. 1F), the total number of eosinophils (*n* = 45,125 eosinophils) also significantly reduced with the disease progression and granuloma involution (Fig. 2F and table S1).

Notably, once inside a mature granuloma, eosinophils were not irregularly scattered as seen in the pre-granulomatous phase (Fig. 1C and fig. S1) but mostly organized in layers (Fig. 2, B and C). To gain a deeper understanding of how the eosinophil population was arranged within granulomas, we next investigated their spatial distribution in more detail. On the basis of the parasite egg localization, three spatial granuloma zones—periovular, medial, and peripheral—(Fig. 2, G, Gi, and Gii) (*31*) were digitally established as detailed (fig. S3), and the eosinophil numbers were quantitated in each zone (fig. S4). Our digital annotation strategy for granuloma zonation helped provide consistent zone measurements even when the section plane showed granulomas with eccentric eggs (fig. S3).

The investigation of zonated individual granulomas (*n* = 424 granulomas, totalizing 1272 annotated zones) revealed that at least 80% of all granuloma types (NE, EP, and P) had a predominant number of eosinophils in the peripheral zone (Fig. 2H). Quantitative analyses demonstrated a significantly greater proportion of eosinophils in the peripheral zone compared to the medial and periovular zones regardless of the type of granuloma and time of infection (Fig. 2, I and J). The least proportion of eosinophils was found in the periovular zone (Fig. 2, I and J). To confirm our findings, we next normalized the eosinophil numbers (total *n* = 45,125) to (i) the zone area (μm^2^) in each granuloma (fig. S5A) and (ii) to the total number of cells in each zone (eosinophil ratio; corresponding to the total number of eosinophils divided by the total number of cells; the equation detailed in the Materials and Methods) (fig. S5, C and D). These two additional analyses significantly demonstrated the dominance of eosinophils in the peripheral zones of all granuloma types (fig. S5, A to D). Last, we applied a spatial positioning analysis (fig. S5, E to G) to study individual eosinophil locations (*n* = 1030 eosinophils) and their relationships to the egg within the granulomas. This strategy captured the eosinophil-egg distances (linear μm), which, after normalization (minimum-maximum), further corroborated the higher presence of eosinophils in the granuloma periphery (fig. S5, H to J).

**Fig. 3.**
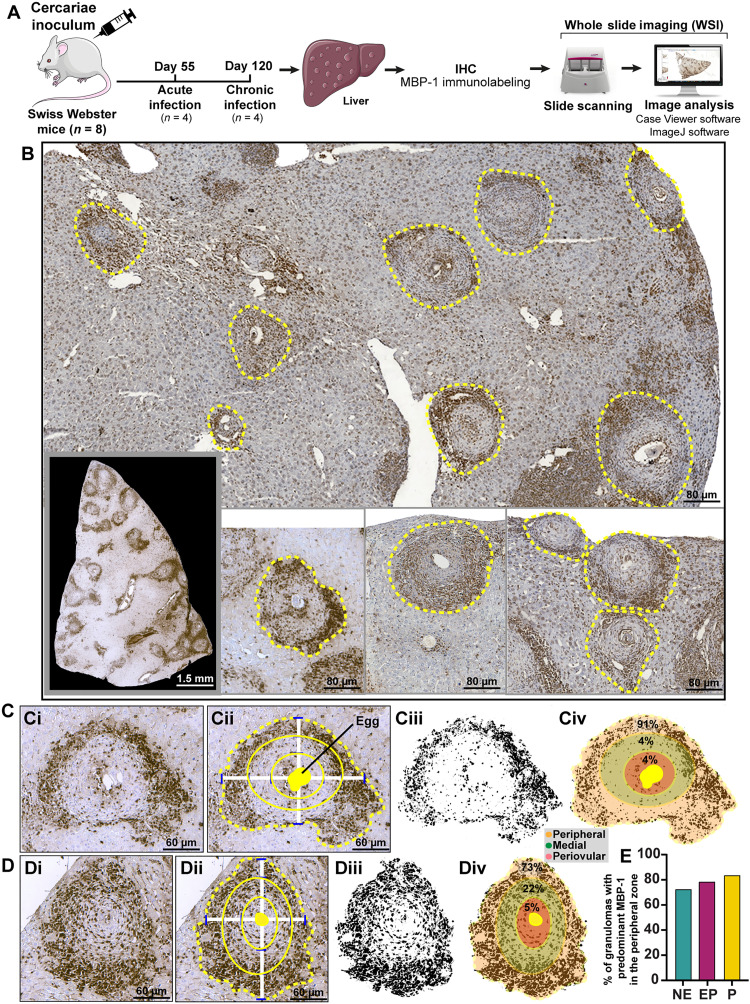
MBP-1 is highly concentrated within the peripheral zone of *Schistosoma* granulomas. (**A**) Experimental setup to investigate MBP-1 immunolabeling within evolutional hepatic granulomas formed in mice with acute and chronic *S. mansoni* infection. Liver sections (*n* = 5 per animal) were processed for IHC using a monoclonal anti-MBP-1 antibody and scanned through WSI. Granulomas were outlined and enumerated, and the immunolabeling intensity was evaluated for determination of the immunostaining area (%). (**B**) Representative WSI scans of the liver after IHC for MBP-1 revealed a high concentration of MBP-1, seen as a brown precipitate, within the peripheral zone of all granuloma types. (**C** and **D**) Zonation approach applied to representative NE (**Ci** to **Civ**) and EP (**Bi** to **Biv**) granulomas for image analysis of the MBP-1 immunolabeling area (%) per zone. A range of 73 to 91% of all immunolabeling was localized in the peripheral zone. Granuloma binary images are shown in (Ciii) and (Diii). (**E**) Quantitative analysis (*n* = 67 granulomas) detected a predominance of MBP-1 staining in the peripheral zone of all granuloma stages.

Classical roles of eosinophils are based on their effector responses associated with the release of their preformed pools of granule-stored products, including major basic protein-1 (MBP-1) (*32*), an abundant eosinophil cationic protein (*33*). MBP is recognized as a classical marker for both eosinophil localization and degranulation (*32*). When released, MBP mediates cytotoxicity to a variety of tissues (*33*, *34*) and is considered an in vitro helminthotoxin against *S. mansoni* larvae (*22*). We next interrogated the spatial distribution of MBP-1 within evolutional granulomas elicited by the acute and chronic experimental infection in mice (Fig. 3A). WSI scans of liver sections showed a robust and spatially well-defined MBP-1 staining in all granuloma types (Fig. 3B). Our zonation approach (fig. S3) applied to individual granulomas demonstrated a high MBP-1 concentration in the granuloma peripheral zone, with eggs negative or showing negligible immunolabeling for this protein (Fig. 3, C and D). Additional quantitative analyses revealed that 73 to 91% of all granulomas (*n* = 67 granulomas) had immunolabeling for MBP-1 predominantly localized in the peripheral zone (Fig. 3E). Thus, immunohistochemistry (IHC) for MBP-1 confirmed the preferential spatial localization of eosinophils in the peripheral zone of all evolutive stages of *Schistosoma* granulomas.

### Eosinophils interact with other immune cells within granuloma peripheral zone

**Fig. 4.**
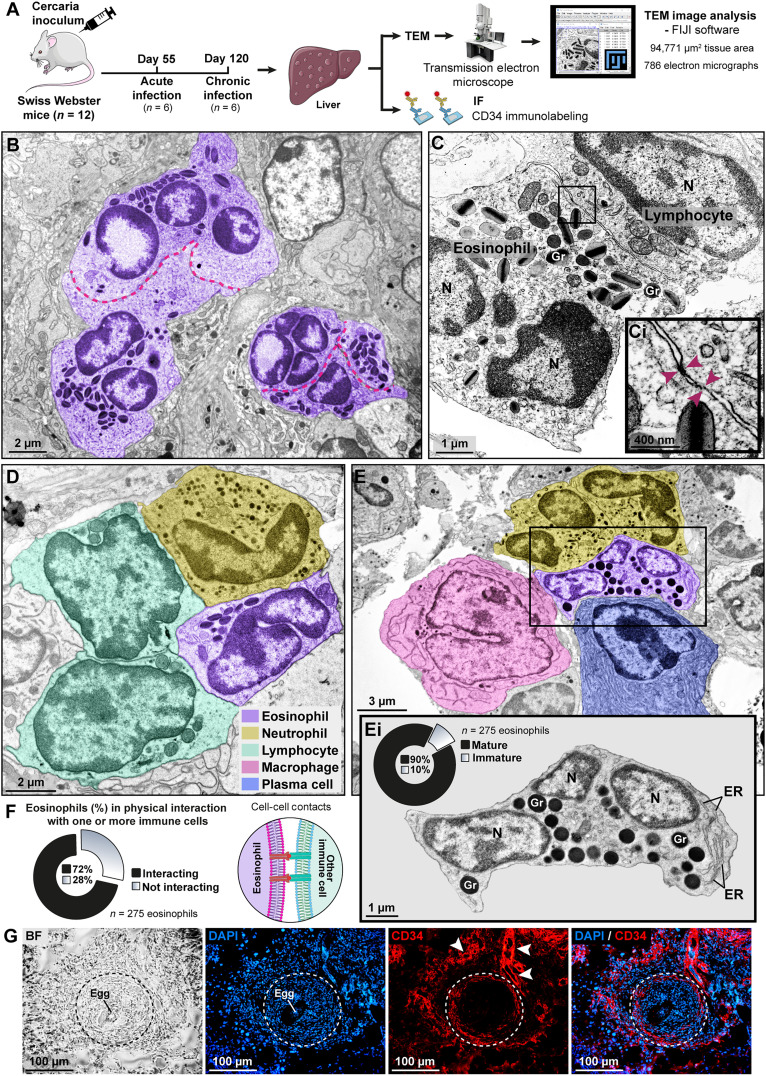
Eosinophils establish physical interaction with other immune cells in the peripheral zone of *Schistosoma* granulomas. (**A**) Experimental setup to investigate the ultrastructure of eosinophils and immunofluorescence (IF) for CD34 cells within the granuloma microenvironment. Hepatic granulomas from both acute and chronic infections were processed for TEM, and electron micrographs obtained from the peripheral zone were analyzed with FIJI software. All intact eosinophils showing entire cell profile (*n* = 275) were scored and individually examined at high resolution. (**B**) Clusters of eosinophils showing cell-cell interactions. Red lines outline the interface between eosinophils. (**C**) Eosinophil-lymphocyte interaction. The boxed area in (C) is shown at higher magnification in (**Ci**). Arrowheads indicate cell-cell contact sites identified by increased electron density. (**D** and **E**) An eosinophil is contacting several types of immune cells. (**Ei**) Higher magnification of the boxed area in (E) shows an incompletely mature eosinophil with coreless granules and a high amount of endoplasmic reticulum (ER) strands. Immature eosinophils corresponded to 10% of all eosinophils visualized in the peripheral zone of granulomas by TEM. (**F**) Most eosinophils (72%) were interacting with one or more immune cells with close apposition of the plasma membranes and presence of connecting points between them as illustrated. N, nucleus; Gr, secretory granule. (**G**) IF for CD34. Representative cryosections of the liver show identical fields of a granuloma (outlined) imaged with bright-field (BF) and fluorescence microscopy for DNA (blue; stained with DAPI) and CD34 (red). Marked positivity was observed in the granuloma peripheral zone and in longitudinal and cross-sectioned blood vessels (arrowheads) surrounding the granuloma. Cells in the medial and periovular zones were negative for CD34. IF data are representative of four animals, 146 granulomas, and three independent experiments.

To identify relevant ultrastructural features of eosinophils while in the granuloma microenvironment, we performed a comprehensive study using TEM. TEM uniquely captures the cell behavior at high resolution in its tissue context, and it is particularly elucidative in demonstrating the intricate and varied secretory activities of eosinophils (*30*). By analyzing a total of 786 electron micrographs from both acute and chronic infections, corresponding to a total area of 94,771 μm^2^ (Fig. 4A), we uncover that eosinophils within the peripheral zone were tightly organized in clusters (Fig. 4B) and notably contacting other immune cells (Fig. 4, C to E). We identified physical interaction between eosinophils and lymphocytes (Fig. 4, C and D), neutrophils (Fig. 4, D and E), macrophages (Fig. 4E), and plasma cells (Fig. 4E). These cellular interactions were characterized by close apposition of the plasma membranes within a narrow nanoscale distance of 15 to 50 nm (mean distance of 27 nm) (Fig. 4, B to E). Moreover, as seen at higher magnification, small areas with increased electron density alongside the plasma membrane interfaces were connecting the two apposing membranes (intercellular contacts) (Fig. 4, C and Ci), a classical ultrastructural feature observed during cellular interactions including between immune cells (*35*, *36*). The increased electron density at specific points of closely apposed plasma membranes likely reflects the local enrichment of adhesion and structural proteins, including receptor and adaptor molecules (*37*). Of 275 eosinophils, 198 (72%) were contacting one or more types of immune cells (Fig. 4F).

Our TEM analyses also revealed that not only mature eosinophils (Figs. 2D and 4, B to D) but also eosinophils with immaturity signs such as the presence of cytoplasmic coreless granules (immature specific granules) and high amount of endoplasmic reticulum strands (*30*, *38*) were present in the peripheral zone of *Schistosoma* granulomas (Fig. 4, E and Ei). These incompletely mature eosinophils corresponded to 10% of the eosinophil population (*n* = 275 cells) (Fig. 4Ei). Eosinophils develop from CD34+ hematopoietic cells, and local, tissue-driven differentiation of CD34+ eosinophil-lineage committed progenitor cells into mature eosinophils has been increasingly demonstrated (*39*–*42*). We then evaluated the presence and distribution of CD34 cells within granulomas. Consistent with our TEM findings, there was marked and dominant immunolabeling for CD34 cells in the granuloma peripheral zone (Fig. 4G) and, as expected, in vascular endothelial cells (Fig. 4G) because CD34 is also expressed by these cells (*43*).

We next investigated the occurrence of ultrastructural changes underlying eosinophil secretion, a process collectively known as degranulation (*30*, *38*). Single-cell imaging of eosinophils (*n* = 272) revealed a prevalent morphologic pattern (78% of all eosinophils) consistent with piecemeal degranulation (PMD), a frequent mode of eosinophil secretion in vivo during eosinophil-associated diseases (EADs) (fig. S6) (*44*). These results were consistent with a previous work in acute schistosomiasis in mice (*25*). PMD, a vesicle-mediated secretory process, which can be precisely detected only with the application of TEM, is involved in the release of small amounts of granule-derived proteins (*38*, *45*, *46*). PMD enables differential secretion of specific cytokines and MBP-1 and has been associated with eosinophil regulatory mechanisms modulating local immune responses (*25*, *32*, *47*).

Together, our findings identify that, within the granuloma peripheral zone, eosinophils organize themselves, differentiate, establish tight interactions with other immune cells, and secrete through PMD, thus supporting a potential immunoregulatory role for these cells rather than direct destruction of parasite eggs.

### The spatial localization of eosinophils is conserved in natural infection

**Fig. 5.**
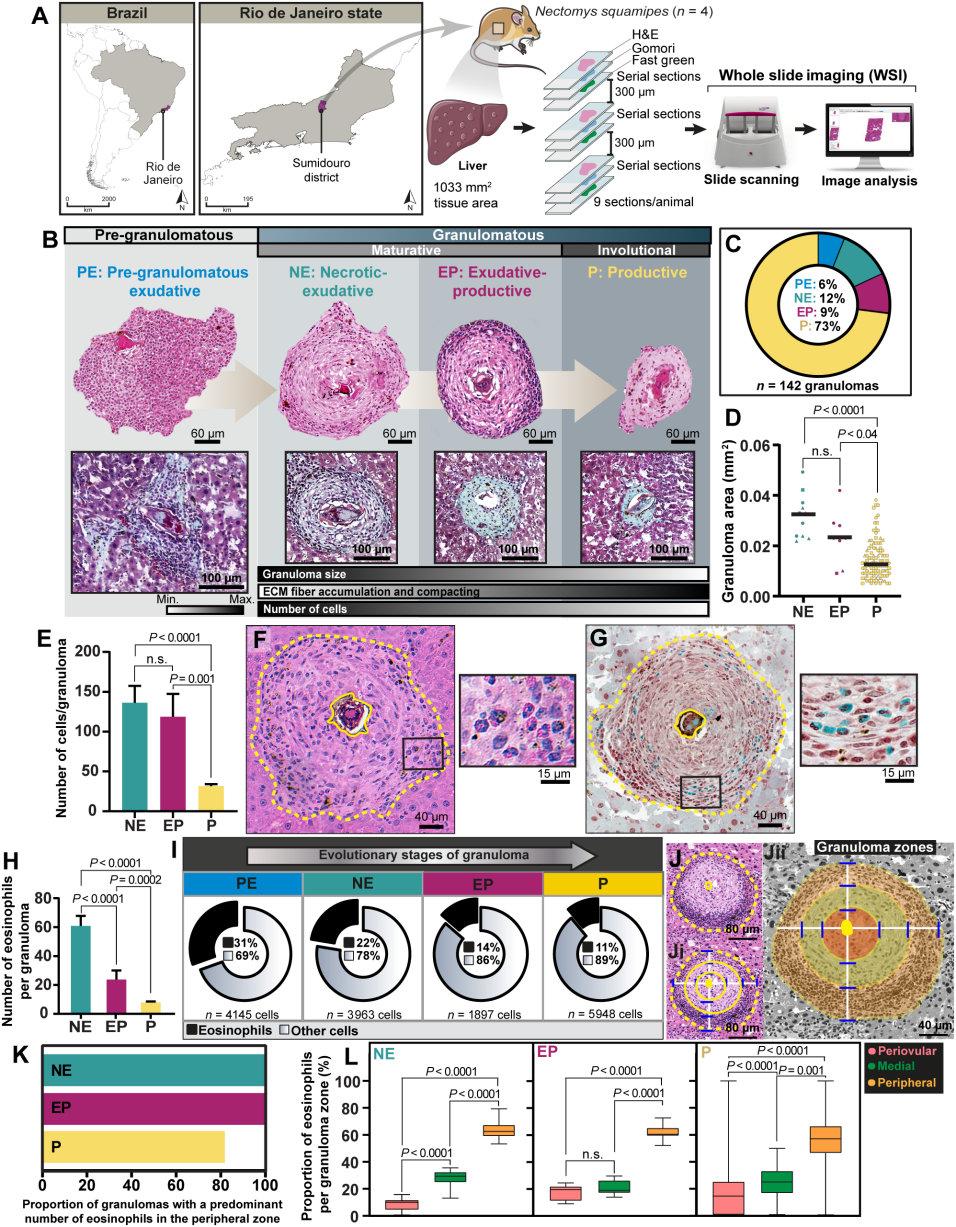
Eosinophils show spatial adaptation within granulomas elicited by natural infection. (**A**) Experimental setup to analyze hepatic granulomas in the wild reservoir *N. squamipes* captured in an endemic area of schistosomiasis in Brazil (Sumidouro district, Rio de Janeiro state). After infection confirmation, tissue collection was performed for histopathology (*n* = 3 sections per animal for each staining) using the same approach as established for the experimental infection in mice. In WSI scans of H&E-stained sections, all granulomas (*n* = 142) were outlined, enumerated, and classified per evolutive stage. (**B** and **C**) Pre-granulomatous and granulomatous phases and their evolutive stages (types and proportions) observed after H&E and Gomori’s staining. Progressive reduction in granuloma size and accumulation of collagen fibers, stained in green, were noted. (**D** and **E**) The granuloma size (area) and its total number of cells were significantly reduced alongside granuloma life. Granuloma area (mm^2^) determined for individual granulomas. Scattered symbols in (D) represent single granuloma data. Different symbols represent individual animals. A total of 15,395 cells were quantified, and the mean number of cells was established/granuloma type. (**F** and **G**) Eosinophils identified by their cytoplasms: pink (H&E) or green (Fast green–Neutral red). (**H**) Number of eosinophils per granuloma type. (**I**) Proportions of eosinophils (*n* = 3402) in relation to the total number of granuloma cells (*n* = 15,395). (**J**) A zonated representative granuloma. (**K**) Eighty to 100% of all granulomas showed most eosinophils in their peripheral zone. (**L**) Eosinophil numbers were significantly higher in the granuloma peripheral zone compared to the medial and periovular zones. Results are expressed as means ± SEM [(D), (E), (H), and (L)]. *P* as indicated by one-way ANOVA followed by Tukey’s multiple comparisons test [(H) and (L) (granuloma NE and EP types)] or Kruskal-Wallis test followed by Dunn’s multiple comparisons test [(D), (E), and (L) (granuloma P-type)].

The consistent eosinophil compartmentalization in the peripheral zone of granulomas from both acute and chronic experimental infection interrogated whether the same pattern occurs in natural infections. Then, we sought to investigate the liver of the neotropical, semiaquatic rodent *N. squamipes* (water rat) (*n* = 4 animals) captured during a long-term study in endemic areas of schistosomiasis in Brazil (Fig. 5A) (*48*). *N. squamipes* plays a crucial role as wild reservoirs for human schistosomiasis, perpetuating the transmission cycle in nature and serving as important biological indicators of the *S. mansoni* transmission sites due to their high susceptibility (*49*). With wide geographic distribution, *N. squamipes* is one of the main nonhuman definitive hosts of *S. mansoni* in Brazil with well-characterized traditional parameters of the infection (consistent worm burden and egg deposition and development of granulomatous responses) (*24*, *49*).

To investigate the histopathology of the liver during the natural infection (*n* = 4 animals), tissue sections (*n* = 3 per animal for each staining) were prepared and digitally scanned (WSI) (Fig. 5A). We used the same approach as for the experimental infection (Fig. 1A), and a total of 142 hepatic granulomas corresponding to 1033 mm^2^ of tissue area were analyzed (Fig. 5A and table S2). The granulomatous inflammatory response of *S. mansoni*–infected *N. squamipes* manifested as well-defined lesions in the liver, as previously reported (*24*, *50*). We found the same evolutive stages of granulomas (Fig. 5B) as observed in the experimental infection (Fig. 1C) but with a higher prevalence of granuloma P-type (Fig. 5C). This may be attributed to the multiple reinfections experienced by this rodent throughout its lifetime, leading to subsequent acquired immunity and/or enhanced capacity to manage the infection (*24*). The presence of recently formed granulomas (PE and NE) informed that parasite eggs continue to reach the liver, likely due to repeated exposure to infectious cercariae, which is also a common aspect in humans living in endemic regions for schistosomiasis (*29*). Evolving granulomas had a progressive accumulation of ECM fibers (Fig. 5B and fig. S2) and reduced cell numbers as the EP granulomas evolved to the P stages (Fig. 5, B, D, and E).

Next, we evaluated the population of eosinophils within granulomas (Fig. 5, F and G) after staining with H&E (Fig. 5, F and Fi) or Fast green–Neutral red (Fig. 5, G and Gi). Our analyses revealed the same pattern of eosinophil accumulation found in the experimental model: initially as a disordered cell collection in the pre-granulomatous phase (PE stage) (Fig. 5B) and subsequently as an organized population within mature granulomas (Fig. 5, F and G). Eosinophil numbers significantly reduced alongside granuloma maturation and involution (Fig. 5H). The proportion of eosinophils (*n* = 3402) in relation to the total number of granuloma cells (*n* = 15,395) was higher in the PE stage (31%) compared to the other stages (range of 11 to 22%) (Fig. 5I).

We next investigated the spatial distribution of eosinophils within individual granulomas (*n* = 142 granulomas, totalizing 426 zones) by applying our zonation approach (Fig. 5J) as detailed in fig. S3. We found that (80 to 100%) of all evolutional granulomas showed predominant numbers of eosinophils in the peripheral zone (Fig. 5K). Quantitative analyses demonstrated a significantly higher proportion of eosinophils in the peripheral zone compared to the medial and periovular zones, with the lower proportions found in the periovular region (Fig. 5L). Application of the same additional quantitative analyses as per experimental infection [eosinophil numbers related to the zone area (μm^2^) in each granuloma (fig. S7A) and eosinophil ratio (fig. S7B)] confirmed eosinophil concentration in the peripheral zone. The spatial positioning analysis of eosinophil locations related to the eggs (fig. S7, C to E) also demonstrated the granuloma periphery as the predominant site for eosinophils (fig. S7, F to H).

Thus, our findings revealed that, within hepatic *Schistosoma* granulomas triggered by natural infection, eosinophils show a “spatial adaptation,” remaining mostly compartmentalized in the granuloma peripheral zone, a conserved localization reproduced, as noted, by the experimental infection.

### Eosinophils occupy a major ecological niche within *Schistosoma* granulomas

**Fig. 6.**
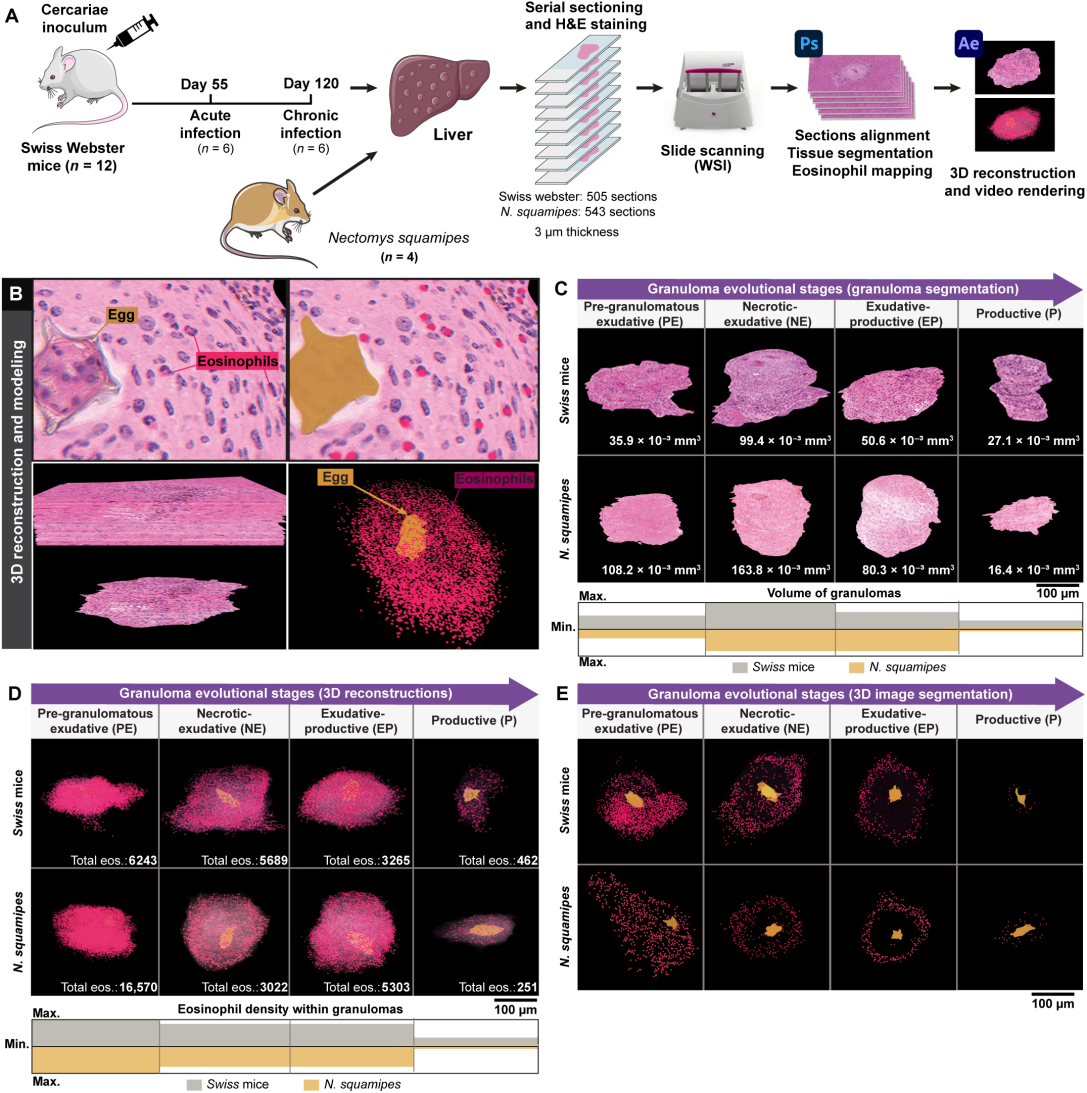
3D reconstructions highlight an eosinophil niche within *Schistosoma* granulomas. (**A**) Experimental setup for 3D reconstruction of serial histological sections of mice and *N. squamipes* hepatic granulomas. Livers from *S. mansoni*–infected animals were embedded in plastic resin (GMA), serially sectioned in a microtome (3 μm thickness), and H&E-stained. In total, 1048 slides, each one containing one section, were scanned (505 from mice and 543 from *N. squamipes*) for whole granuloma identification and segmentation. Serial sections were aligned and labeled with the use of Adobe Photoshop software. 3D reconstruction and rendering were performed with Adobe After Effects software. (**B**) Steps for 3D reconstruction and modeling of a representative granuloma as seen in movie S1. The *Schistosoma* egg (yellow) and eosinophils, identified by their typical morphology (segmented nuclei and acidic cytoplasm stained in pink with H&E as in Fig. 2B were manually labeled red). Sections were then sequentially organized in the *z* axis to form a 3D structure. The granuloma was segmented from the hepatic tissue to reveal the spatial distribution of its eosinophil population within the granuloma microenvironment. (**C**) Reconstruction of segmented granulomas of all evolutional stages from both infection models. (**D**) 3D spatial localization of eosinophils within the granuloma evolutional stages. (**E**) Interior of the 3D reconstructed granulomas after segmentation. Although eosinophils appear as a disordered cell collection in the pre-granulomatous phase (PE stage), the preferential localization of eosinophils is uncovered alongside the granulomatous stages (NE, EP, and P). The reconstructions of entire granulomas for each evolutive stage (PE, NE, EP, and P) are also shown in movie S2 (experimental infection) and movie S3 (natural infection).

To further confirm the eosinophil spatial organization within the hepatic granulomas triggered by *S. mansoni* infection, we performed quantitative 3D histological reconstructions of entire granulomas (Fig. 6 and table S3). A representative granuloma of each evolutional stage (PE, NE, EP, and P) from both experimental and natural infections was fully sectioned. After alignment and segmentation, eosinophils were mapped in each serial section through WSI before granuloma reconstruction (Fig. 6, A and B, and movie S1). With our unique approach, we calculated the volume of each granuloma type (Fig. 6C) and observed the cumulative presence of eosinophils across the granuloma volume, spatially separate from all other cells and ECM fibers (Fig. 6D). Consistent with our previous data here, both volume (Fig. 6C) and the number of eosinophils (Fig. 6D) decreased during the process of granuloma formation (table S3).

The entire eosinophil population within 3D-reconstructed granulomas is shown in Fig. 6D and after 3D image segmentation (Fig. 6E) for better visualization of the eosinophil spatial localization. Both experimental (movie S2) and natural (movie S3) infections showed the same pattern of 3D spatial compartmentalization of eosinophils throughout the granuloma life (Fig. 6D). The pre-granulomatous phase (PE stage) was visualized as a very dense and disordered collection of recruited eosinophils (Fig. 6, D and E). However, within established granulomas, eosinophils organized themselves within the peripheral zone (Fig. 6, D and E).

Together, eosinophils predominantly inhabit a distinct, peripheral region within evolving *Schistosoma* granulomas, thus delineating an ecological niche conserved across time and space and with functional implications to the cell community and granuloma life.

## DISCUSSION

Although the traditional view of eosinophils is that these cells kill parasitic helminths like *S. mansoni* as part of an innate immune response, the massive presence of eosinophils within *S. mansoni* egg–containing granulomas remains an enigma (*8*, *9*). As one of the most extensive studies of eosinophils across evolving *Schistosoma* granulomas to date, we identified an ecological niche of eosinophils conserved in both experimental (acute and chronic) and natural infections. Quantitative image analyses, integrated with a zonation approach applied to individual granulomas and 3D reconstructions of entire granulomas, revealed that most eosinophils lodge in a particular territory within the granuloma boundaries.

Spatially in this niche, eosinophils establish solid interactions with other immune cells and release their granule-stored products mainly through PMD. Yet, not only fully mature but also incompletely mature eosinophils inhabit this niche. Therefore, the term “ecological niche,” meaning a specific microenvironment in which cells can settle, differentiate, and interact with other cell populations (*8*), can be fully applied to the uncovered eosinophil spatial compartmentalization. This work contributes to an expanding understanding of *Schistosoma* granulomas as locations in which inflammatory cells—eosinophils—are not merely concentrated but spatially adapted to the microenvironment.

Our findings highlight the robustness and reliability of the eosinophil ecological niche across two distinct infection conditions. At least 80% of all evolutional mature granulomas, individually analyzed in both experimental (acute and chronic) and natural infection models, showed a predominant number of eosinophils within this niche. This is particularly noteworthy due to the notable differences inherent to experimental and natural infections (*51*, *52*). Experimental murine infections involve a single exposure with defined *S. mansoni* isolates, time, and dose of infection, whereas natural infections are gradually acquired with natural isolates of the parasite and undefined time of infection (*51*). Most *Schistosoma* infections in humans are acquired gradually over years (*53*). Moreover, as noted by our study and others, natural infections are less intense than the experimental ones (*51*, *52*). Here, we detected that, in the natural infection, several parameters were reduced compared to the experimental one (tables S1 and S2), including (i) the mean number of parasite eggs per hepatic tissue area, (ii) the mean number of mature granulomas (NE and EP) per tissue area, and (iii) the total number of cells and eosinophils within granulomas. On the other hand, both the proportion and number per tissue area of granulomas P, which is the involutional type of granulomas, is much higher in the water rats (Fig. 5C and table S2). These findings highlight the notable physiological adaptation of the wild reservoir to the parasite (*24*, *54*) while sustaining an ecological niche of eosinophils within *Schistosoma*-induced granulomas.

What is the functional meaning of an “eosinophil ecological niche” in hepatic granulomas triggered by schistosomiasis mansoni? Our findings demonstrate that, although eosinophils are irregularly distributed in a pre-granulomatous phase (PE stage), they are very organized within mature evolutional granulomas in a way that could maximize their effectiveness to the granuloma life. As a potential result of such an organization, eosinophils can more efficiently communicate with other cells. Our TEM quantitative analyses at single-cell resolution captured that 72% of eosinophils in this niche interacted physically with other cells of the immune system, particularly lymphocytes, neutrophils, plasma cells, and macrophages. Cell-cell contact is an important event underlying the functioning of immune cells within environmental niches and considered critical for the development of efficient immunoregulation (*37*). Eosinophils communicate with a variety of immune cells (*55*) and contain an array of molecules, which potentially enable them to interact with every cell type in a tissue (*56*). As a source of cationic proteins such as MBP-1 and many cytokines, eosinophils can differentially and gradually release these granule-derived immune mediators through PMD (*32*, *46*, *57*), a secretory process confirmed here during schistosomiasis (*25*) that may likewise reflect their role as immunoregulatory cells (*25*, *47*). Thus, our findings illuminate a regulatory activity for eosinophils, which may be modulating local tissue immune responses in schistosomiasis.

In addition to cell-cell interactions, in the light of Ecology, there are microenvironmental factors that can govern cell survival and differentiation within an ecological niche (*8*). Thus, the spatial architecture of the eosinophil ecological niche may benefit eosinophil differentiation, maturation, and survival in situ and eosinophils have a collection of cell surface receptors crucial for their own survival (*56*, *57*). We provided evidence that immature eosinophils and their progenitors, represented by CD34+ cells, occupy this niche. In line with our findings, a high number of eosinophils in different stages of maturation was also localized in the peripheral zone of hepatic *Schistosoma* granulomas in mice (*58*). Spatial compartmentalization and local maturation have recently emerged as important features of eosinophil biology, acting as key contributors to eosinophil functional diversity (*59*, *60*).

Deciphering in detail the distribution, behavior, and specific functions of eosinophils within granulomas is of considerable interest to understanding schistosomiasis. This has been a challenging task, especially because of the complex architecture and dynamics of *Schistosoma* granulomas. Although eosinophilia is traditionally linked to the in vivo destruction of *Schistosoma* eggs in hepatic granulomas (*61*–*63*), eosinophil-deficient mouse models (*64*–*67*) or mice treated with a monoclonal antibody anti–interleukin-5, an essential cytokine for eosinophil development (*68*), showed no impact on worm or tissue egg burdens. The absence of eosinophils does not seem to affect the granuloma formation either. Most works using eosinophil depletion did not find changes in granuloma size and number (*64*, *65*, *68*) or fibrosis development (*65*, *68*), although reduced fibrosis (*66*, *67*), as well as larger (chronic phase) (*66*) or smaller (in both acute and chronic phases) (*67*) granulomas were also reported. However, the comparability of these multiple studies is challenging, considering that they did not account for the evolutive stages of granulomas. Our analyses show that the *Schistosoma* granuloma is an evolutionary system that reduces in size, total number of cells, and number of eosinophils alongside granuloma maturation.

Our findings do not favor a direct role for eosinophils toward the parasite. The segregation of most eosinophils and their MBP-1 content in the peripheral zone of evolutional granulomas, and not within their periovular zones, in which the parasite eggs are biologically active and secreting SEAs, supports a scenario inconsistent with the eosinophil view as “helminth killers” and aligned with a more recent perspective that emphasizes their prominent role as regulators of immune responses (*9*, *55*, *56*, *69*). The discovery of a conserved eosinophil ecological niche within *Schistosoma* granulomas contributes to the understanding of the complex eosinophil biology in helminth infections and highlights the intricate eosinophil-parasite-microenvironment interactions established during very long coevolution.

## MATERIALS AND METHODS

### Animals and models of infection

#### Mice

Swiss Webster female mice were obtained from the Federal University of Minas Gerais (UFMG) animal facility (Belo Horizonte, Brazil) and maintained under standard conditions. Animals aged 70 days were inoculated (*n* = 12) or not (*n* = 6) with a single inoculum of *S. mansoni* cercariae (100 cercariae per mouse), LE strain, as previously described (*70*). Animals were infected by an experienced technician at the Laboratory of Schistosomiasis (Department of Parasitology, UFMG, Brazil) where the LE strain of *S. mansoni* has been routinely maintained through successive passages in *Biomphalaria glabrata* and *Mesocricetus auratus* as previously described (*71*). The infection was confirmed by the presence of parasite eggs in the animal feces in the fifth week of infection (*25*). All classical parameters confirmatory of a fully developed acute infection, such as hepatomegaly, high density of eggs in the liver, well-characterized granulomatous inflammation around deposited eggs, alteration of liver enzymes, increased levels of T_H_2 cytokines and extensive eosinophil infiltration have been consistently demonstrated in this model (*24*, *25*). We have complied with all relevant ethical regulations. Animal studies were approved by the Oswaldo Cruz Foundation Ethics Committee on Animal Use [CEUA (Comissão de Ética no Uso de Animais)/FIOCRUZ, protocol LW-32/2012], which follows the Brazilian guidelines recommended by the National Council for Animal Experimentation–CONCEA (Conselho Nacional de Controle em Experimentação Animal). All animals were monitored daily for survival and well-being (home cage evaluation, body condition, skin lesions, mobility, and other general conditions) (*72*).

#### Nectomys squamipes

The neotropical, semiaquatic *N. squamipes*, also known as water rat, is a natural host for *S. mansoni* in Brazil with a wide geographical range (*49*, *73*). *N. squamipes* specimens were captured in an endemic area (Sumidouro district, Rio de Janeiro state, Brazil) by the technical staff from the Laboratory of Biology and Parasitology of Wild Mammals Reservoirs (FIOCRUZ, Rio de Janeiro, RJ, Brazil) as previously described (*48*, *49*) and under authorization of the Brazilian Government’s Chico Mendes Institute for Biodiversity and Conservation (ICMBIO, license 13373). According to routine procedures, adult rodents were assessed to confirm infection by *S. mansoni* and other parasites and divided into two groups (infected and noninfected; *n* = 4 per group). Infected *N. squamipes* were identified by the presence of adult worms in mesenteric veins using perfusion of the portal-hepatic system (*48*). Worms recovered from each infected animal were counted with the aid of a stereomicroscope. In addition to the presence of adult worms in the mesenteric veins, positivity was confirmed by parasite eggs found in stool tests (*74*). All procedures followed the guidelines for the capture, handling, and care of animals of the Ethical Committee on Animal Use of the Oswaldo Cruz Foundation (CEUA, licenses LW81/12 and L-036/2018). Biosafety procedures and personal protective equipment, including biosafety level three respirators, were used during all procedures involving animal handling and biological sampling.

#### Organ harvest

Mice were euthanized on day 55 (*n* = 6) and 120 postinfection (*n* = 6), corresponding to the acute and chronic phases of the disease, respectively, by exsanguination (full bleed) under deep anesthesia by cardiac puncture as before (*25*). *N. squamipes* specimens were euthanized by exsanguination (full bleeding) under deep anesthesia through cardiac puncture with perfusion of the portal-hepatic system as before (*48*). The anesthetic protocols included ketamine (100 mg/ml) combined with acepromazine (10 mg/ml) at a ratio of 9:1 (dose of 0.15 mg;/100 g body weight) (*75*). Tissue collection for *N. squamipes* was performed at the expedition site.

### Specimen processing for histological analyses

Liver samples from infected and uninfected animals were removed from the right lobe and divided into ~5-mm^3^ fragments, which were immediately fixed in 4% paraformaldehyde in buffered phosphate, pH 7.4, 0.1 M overnight at 4°C (*25*). Next day, the specimens were transferred to the same buffer solution and kept at 4°C for further histological processing. Samples were embedded in GMA (Leica Historesin Embedding Kit, Leica Biosystems, Heidelberg, Germany) for histopathological evaluation or Paraplast (Sigma-Aldrich, USA) for IHC as described below. For the study of histopathological parameters, samples were dehydrated, embedded in GMA and cut at 3 μm thickness using a microtome (Leica RM2155) (*25*). One advantage of this resin is to avoid tissue damage induced by heating required for the embedding step with classical paraffin embedding. Moreover, GMA provides higher tissue resolution, important to visualize fine morphological details. Better resolution is due to plastic polymerization that causes less shrinkage and retraction compared to conventional techniques (*25*). Nine tissue sections were obtained per animal. Each group of three sections was serially acquired and stained respectively with H&E, Gomori’s trichrome stain (Sigma-Aldrich) and Fast green–Neutral red (Sigma-Aldrich). An interval of 300 μm, as illustrated in fig. S1B, was kept between each group of three sections to ensure analysis of different granulomas, thus precluding reanalysis of the same granuloma (*25*). Sections stained with H&E were used for qualitative and quantitative evaluation of the granulomatous inflammation, whereas Gomori’s trichrome stain was applied for visualization and quantification of ECM fibers (fibrosis) (*24*). Sections stained with Fast green–Neutral red were used to confirm eosinophil distribution. Eosinophils were detected in histological sections by their typical features: segmented nuclei, which stain purple (hematoxylin) or red (Neutral red) and highly acidophilic cytoplasm due to the presence of a rich population of secretory granules, which stain pink (eosin) (*24*, *25*) or green (Fast green) (*76*).

### IHC for MBP-1

IHC was performed using a detection system (Novolink Polymer Detection Kit, Leica Biosystems, Wetzlar, Germany), and a rat monoclonal anti-mouse IgG2a MBP-1 primary antibody (clone 14.7.4, 5 μg/ml, Lee Laboratories, Mayo Clinic, Scottsdale, AZ, USA) whose MBP-1 specificity has been well validated in previous studies (*25*, *77*). In brief, Paraplast-embedded 5-μm–thick sections from mouse livers (*n* = 4 animals per group) were deparaffinized, hydrated, washed 1x in phosphate-buffered saline (PBS), pH = 7.4, and submitted to antigen retrieval with sodium citrate buffer, pH = 6.0, moist heat, and pressure prior to immunolabeling (*78*). After blocking endogenous peroxidase and unspecific proteins with hydrogen peroxidase blocking solution and protein block solution (Novolink), respectively, cells were incubated overnight at 4°C with the primary antibody. Next, cells were incubated in a solution containing the secondary antibody [rabbit anti-mouse IgG, <10 μg/ml in 10% (v/v) animal serum in 0.1% tris-buffered saline, ProClin 950, Novolink], a polymer [anti-rabbit Poly-HRP-IgG, <25 μg/ml, in 10%, (v/v), animal serum in 0.1% tris-buffered saline, ProClin 950; Novolink], and the diaminobenzidine chromogen (DAB) diluted in a substrate buffer with ≤0.1% hydrogen peroxide and preservative (Novolink Kit) for reaction visualization. For negative controls, a rat IgG2a kappa isotype control (R35-95, BD Biosciences) was used as a primary antibody to verify the specificity of the reaction. Positive staining was detected by brown-colored products in the cytoplasm.

### Immunofluorescence for CD34

After fixation as above, samples were immersed in cryoprotectant (30% sucrose in PBS) overnight at 4°C, embedded in Tissue-Tek O.C.T. compound, and sectioned on a cryostat (5 μm thickness) at −25°C. Tissue sections were allowed to air-dry for 15 min at room temperature (RT), washed with PBS, and blocked with 50 mM ammonium chloride (NH_4_Cl) for 30 min, followed by a solution containing 2% skim powdered milk, 3% bovine serum albumin (BSA), and 8% fetal bovine serum for 40 min. Additional blocking steps with 3% BSA and 1% BSA solutions were performed for 15 min each. This was followed by an overnight incubation at 4°C with an anti-CD34 (sheep anti-mouse) affinity-purified polyclonal antibody (R&D Systems, catalog no. AF6518) or with the corresponding irrelevant antibody (Thermo Fisher Scientific, catalog no. 31243) at 2 μg/ml diluted in 1% BSA. After washing with PBS and sequential blocking with 3% and 1% BSA, sections were incubated for 2 hours at RT with the secondary antibody donkey anti-sheep Alexa Fluor 594 (Thermo Fisher Scientific, catalog no. A-21098) at 5 μg/ml in 1% BSA (1:400 dilution). Sections were counterstained with 4′,6-diamidino-2-phenylindole (DAPI; Thermo Fisher Scientific, catalog no. 62248) for visualization of nuclei and mounted using ProLong Gold Antifade mounting medium (Thermo Fisher Scientific, catalog no. P36934) for visualization under a fluorescence microscope (BX-60, Olympus, Melville, NY, USA). At least three sections were analyzed per animal.

### Transmission electron microscopy

Samples for TEM were prepared as before (*25*). Liver fragments containing granulomas were fixed in a mixture of 1% paraformaldehyde and 1.25% glutaraldehyde [EM grade, 50% aqueous, Electron Microscopy Sciences (EMS), Hatfield, PA, USA] in 0.1 M PBS, pH = 7.4, at RT. After 2 hours, fragments were sliced into smaller pieces (1 mm^3^) and fixed in the same fixative solution described before overnight at 4°C. Then, fragments were washed twice in 0.1 M sodium phosphate buffer for 4 hours each at 4°C and kept in the same buffer for further processing. Postfixing was done in 1% osmium tetroxide in Sym-Collidine buffer, pH 7.4, for 2 hours at RT, followed by washing with sodium maleate buffer, pH 5.2. Samples were stained en bloc in uranyl acetate 2% (EMS) in 0.05 M sodium maleate buffer, pH 6.0, for 2 hours at RT and washed in the same buffer as before. Then, samples were dehydrated in graded ethanol and acetone and infiltrated and embedded with a propylene oxide–Epon sequence (Eponate 12 Resin; Ted Pella, Redding, CA, USA). Polymerization happened at 60°C for 16 hours. Ultrathin sections were done with a Leica ultramicrotome (EM UC6), placed on copper grids (Ted Pella), stained with lead citrate, and examined with a transmission electron microscope (Tecnai G2-20, Thermo Fischer Scientific/FEI 2006, Eindhoven, The Netherlands) at 80 to 120 KV equipped with an MT NanoSprint43-MkII (43 megapixel) charge-coupled device camera. At least three grids from each sample were analyzed.

### Image analysis

#### Whole slide imaging (WSI)

Slides prepared for histological and IHC analyses were scanned using a 3DHistech Panoramic Scan Digital Slide Scanner (3DHistech Ltd., Budapest, Hungary), which enables a resolution of 0.23 μm per pixel, connected to a computer (Fujitsu Technology Solutions GmbH, Munich, Germany) (*28*). Tissue section areas were analyzed using Pannoramic Viewer 1.15.2 SP2 RTM (3DHistech) software and Histoquant (3DHistech) module, which provide a detailed morphometric analysis with precise measurements of different morphological parameters as described below (*24*, *28*).

#### Enumeration and classification of granulomas

Hepatic H&E-stained sections, obtained as above through WSI, were measured, and all evolving granulomas (total *n* = 442 from mice and *n* = 142 from *N. squamipes*) were outlined by hand, identified with a number, and automatically enumerated using the Panoramic Viewer. Granuloma identification was validated by three experienced researchers trained in experimental histopathology of schistosomiasis mansoni and image analysis. Granulomas lacking the egg were not included. The following morphometric parameters were evaluated: (i) enumeration and classification of each granuloma per evolutionary stage as detailed in fig. S1, (ii) frequency of each granuloma evolutionary stage, (iii) size (area) of individual granulomas, and (iv) total number of cells within each granuloma. In parallel, Gomori’s trichrome–stained sections subjacent to the H&E-stained sections, were inspected for visualization of ECM deposition and organization, which is helpful for the granuloma classification (*24*). ECM fibers were quantitated as below.

#### Quantification of ECM fibers

Gomori’s trichrome–stained sections were digitized at 20× magnification using the 3DHistech scanner as above and analyzed using the Case Viewer 2.3 software (Budapest, Hungary). The total area and the area fraction of the ECM were measured by semiautomatic morphometric analysis, using the ImageJ 1.53e software (National Institutes of Health, USA). We applied a Gomori mask that allowed the automatic calibration of green pixels from Gomori-stained regions to create a binary image as shown in fig. S2. The threshold of 0 to 170 pixels was applied to calculate the area proportion of the Gomori staining. A total of 240 granulomas [120 for the experimental infection (acute + chronic) and 120 for the natural infection; *n* = 40 per each granuloma type (NE, EP, and P)] were analyzed.

#### Eosinophil quantification and distribution within evolutional granulomas

To identify and quantitate the number of eosinophils within each granuloma, high-resolution images from H&E-stained sections (*n* = 3 sections per animal) of individual granulomas (total *n* = 442 from mice and *n* = 142 from *N. squamipes*) were acquired through WSI. Eosinophils were then carefully inspected by trained researchers in eosinophil cell biology and histopathology of schistosomiasis, identified based on their typical histological features [segmented nuclei, which stain purple by hematoxylin and highly acidophilic cytoplasm, which stain pink by eosin (*24*, *25*)], and manually annotated using Histoquant module. All other cells clearly distinct from eosinophils within the granuloma were manually identified based on the presence of a hematoxylin-stained nucleus and a clear cytoplasm. Annotated cells were automatically quantitated by Histoquant module. The specific quantification procedures for eosinophils are presented step-by-step in fig. S4. The following parameters were evaluated for each granuloma: (i) total number of eosinophils and (ii) proportion of eosinophils related to the total number of cells.

#### Eosinophil quantification within granuloma zones and zonation approach

The numbers and spatial distribution of eosinophils were also evaluated per granuloma region, specifically within three different zones in each granuloma: periovular, medial, and peripheral (*31*, *58*). To reliably establish these zones, we developed a strategy—zonation approach—which was applied to each individual granuloma. The digital annotation workflow for granuloma zonation is detailed in fig. S3. Granulomas were first outlined (fig. S3, Ai and Bi), and two lines were drawn in a perpendicular plane intersecting at the center of the egg (fig. S3, Aii and Bii). Note that one line must pass through the major axis of the egg. Then, along these lines, the distance from the eggshell to the granuloma boundary was divided into three equal parts. Each line was subdivided into three equal segments, with division points marked along the length of the lines. The division points on the two lines were then connected, resulting in the formation of three concentric circles, which ultimately established the three zones: periovular, medial, and peripheral (fig. S3, Av and Bv). Thus, these zones were reliably identified in granulomas with either central (fig. S3, Av and Avi) or eccentric (fig. S3, Bv and Bvi) eggs. The area of each zone, the number of eosinophils, and the number of other cells were established in each granuloma zone. The proportion of eosinophils and the number of eosinophils per area (μm^2^) were also determined per zone. Last, the eosinophil ratio (*R*_eos_) was calculated for each granuloma zone as below (Eq. 1), where *N*_eos_ is the number of eosinophils and *N*_cells_ is the number of other cells. A total of 424 granulomas, totalizing 1272 annotated zones for mice and 142 granulomas totalizing 426 zones for *N. squamipes* were analyzed

$$R_{\text{eos}}={\text{log}}_{10}\left(\frac{N_{\text{eos}}}{N_{\text{cells}}}\times 100\right)$$ (1)

#### Eosinophil spatial positioning and distance to granuloma egg

WSI high-resolution images of segmented granulomas with annotated eosinophils, obtained with the Panoramic Viewer software as shown in figs. S5E and S7C, were used to evaluate the spatial positioning and distance of individual eosinophils to the eggs (figs. S5, F to J, and S7, D to H). Analyses were performed by imaging-based quantitative analyses using Fiji software (ImageJ, National Institutes of Health, USA). First, each image scale was set (Analyze/Set Scale) and the granuloma egg was manually marked. Then, the *X* and *Y* spatial coordinates of the egg were obtained using the Centroid measurement (Analyze/Set Measurements). Sequentially, the granuloma images were smoothed to prevent noise detection (Process/Smooth) and segmented by applying a threshold (Image/Adjust/Color Threshold) to the image. This process separates the foreground signal from the background signal thus selecting the region-of-interest—eosinophils—each one imaged as an individual point (figs. S5F and S7D). Last, the “Analyze Particles” tool (size: 1 μm to infinity; circularity: 0.90 to 1.00) was used to assess the *X* and *Y* spatial coordinates of eosinophils (figs. S5G and S7E). The data were then tabulated and the distance (*d*) between each eosinophil from the center of the egg within the granuloma was determined by applying the Euclidean distance equation (Eq. 2), where *X*_1_ and *Y*_1_ were the coordinates of the egg and *X*_2_ and *Y*_2_ were the coordinates of the eosinophil

$$d=\sqrt{\left\{{\left(x_{2}-x_{1}\right)}^{2}+{\left(y_{2}-y_{1}\right)}^{2}\right\}}$$ (2)

In total, 1030 eosinophils for the experimental infection and 750 eosinophils for the natural infection were evaluated as above. In addition, for each granuloma type (NE, EP, and P), the eosinophil-egg distances were normalized from 0 to maximum, according to Eq. 3, where *X*_min_ and *X*_max_ are the minimum and maximum distances recorded. As a result, the 0-distance value is the nearest eosinophil to the egg. Then, three equal distance ranges were determined for each type of granuloma and the proportions of eosinophils were calculated for each range (figs. S5, H to J, and S7, F to H). The linear distribution of eosinophils in different distances was plotted (figs. S5, H to J, and S7, F to H, bottom panels), and the mean distance between the egg center and the first eosinophil was acquired for each type of granuloma

$$X\prime =\frac{X-X_{\text{min}}}{X_{\text{max}}-X_{\text{min}}}*X_{\text{max}}$$ (3)

#### IHC quantitative analysis

The slides were digitized using the 3DHistech scanner (Budapest, Hungary), and WSI scans were analyzed at 20× magnification using the Case Viewer 2.3 software (Budapest, Hungary). The total area and the area fraction corresponding to the density of the MBP-1 immunolabeling were measured by semiautomatic morphometric analysis using the ImageJ 1.53 software (National Institutes of Health, USA). The hematoxylin/DAB mask was selected, and the brown pixels from DAB-stained cells were automatically calibrated to create a binary image. The threshold of 0 to 132 pixels was applied to calculate the proportion of the immunostaining.

#### TEM qualitative and quantitative analyses

Electron micrographs (*n* = 786) show that infiltrated eosinophils were randomly acquired at magnifications ranging from 1200× to 12,000×, and the total of 94,771 μm^2^ of tissue area from the peripheral zones of hepatic granulomas was analyzed. To investigate the eosinophil ultrastructure, all eosinophils found in the tissue areas were scored (*n* = 296) and the cells were evaluated for maturity, immaturity, and secretory aspects as previously described (*30*) as well as their cell-cell interactions. For immaturity and cell interaction quantitative analyses, the number of lytic eosinophils (*n* = 21) was excluded. In tissue areas showing cellular interactions, the number of eosinophils in physical interaction with other immune cells (cell-cell contacts) was established and the distance between the two apposing plasma membranes was measured in five different points encompassing the length of the interaction area. All quantitative analyses were performed using Fiji software platform (ImageJ, National Institutes of Health, USA).

#### 3D reconstructions of serially sectioned hepatic granulomas

3D reconstructions of entire granulomas were adapted from a previous work (*79*) using serial 2D images obtained from the livers through WSI. Briefly, GMA-embedded 5-mm^3^ tissue blocks from both experimental (mice) and natural (*N. squamipes*) models of *S. mansoni* infections were serially cut at 3 μm on a microtome (Leica RM2155). In total, 505 serial sections were obtained from Swiss mice and 543 from *N. squamipes*. The sections were stained with H&E and scanned on a digital scanner as above. Representative granulomas (one for each evolutionary stage) were identified and serial images in TIFF format were captured for each granuloma type from the same animal based on alignment with Adobe Photoshop software (Adobe Inc., San Jose, CA, USA). Next, infiltrating eosinophils in different regions of the granuloma and the parasite egg were identified as described above and highlighted in each serial-aligned image. The images were exported (PNG format) and sequentially imported into Adobe After Effects software (Adobe Inc.) to construct the 3D volumes. Last, animations were made using object rotation tools in the three spatial axes (*x*, *y*, and *z*) and changes in layer opacity, position, and scale for a more complete visualization of the reconstructed tissue and eosinophil spatial localization.

### Statistical analysis

Statistical analyses were performed and graphs were made using GraphPad Prism software, version 8.0.2 (San Diego, CA, USA). For two-group comparisons, the Student’s *t* test was applied for parametric variables, and the Mann-Whitney test was applied for nonparametric variables. For three or more comparisons, the one-way or two-way analysis of variance (ANOVA) test was performed, followed by Tukey’s post hoc test for parametric variables, or the Kruskal-Wallis test followed by Dunn’s multiple comparisons test for nonparametric variables. The level of significance used for all analyses was ≤0.05. Data are presented as the means ± SEM unless otherwise specified. Data in bar plots graphs are expressed as means or means ± SEM. Data in box plot graphs are presented as minimal to maximal values and median.
