# Supplementary material for: A major ecological niche of eosinophils in evolving Schistosoma granulomas challenges the eosinophil view as “helminth killer” cells
Source: Sci Adv. 2025 Jun 11;11(24):eadt2779. doi: 10.1126/sciadv.adt2779 (PMC12154175; doi:10.1126/sciadv.adt2779)
Supplement: Supplementary file 1 — Figs. S1 to S7 Tables S1 to S3 Legends for movies S1 to S3 [file sciadv.adt2779_sm.pdf]

Science Advances
AAAS

Supplementary Materials for

A major ecological niche of eosinophils in evolving *Schistosoma* granulomas challenges the eosinophil view as "helminth killer" cells

Luccas M. Barata *et al.*

Corresponding author: Rossana C. N. Melo, rossana.melo@ufjf.br

Sci. Adv. **11**, eadt2779 (2025)
DOI: 10.1126/sciadv.adt2779

The PDF file includes:

Figs. S1 to S7
Tables S1 to S3
Legends for movies S1 to S3

Other Supplementary Material for this manuscript includes the following:

Movies S1 to S3

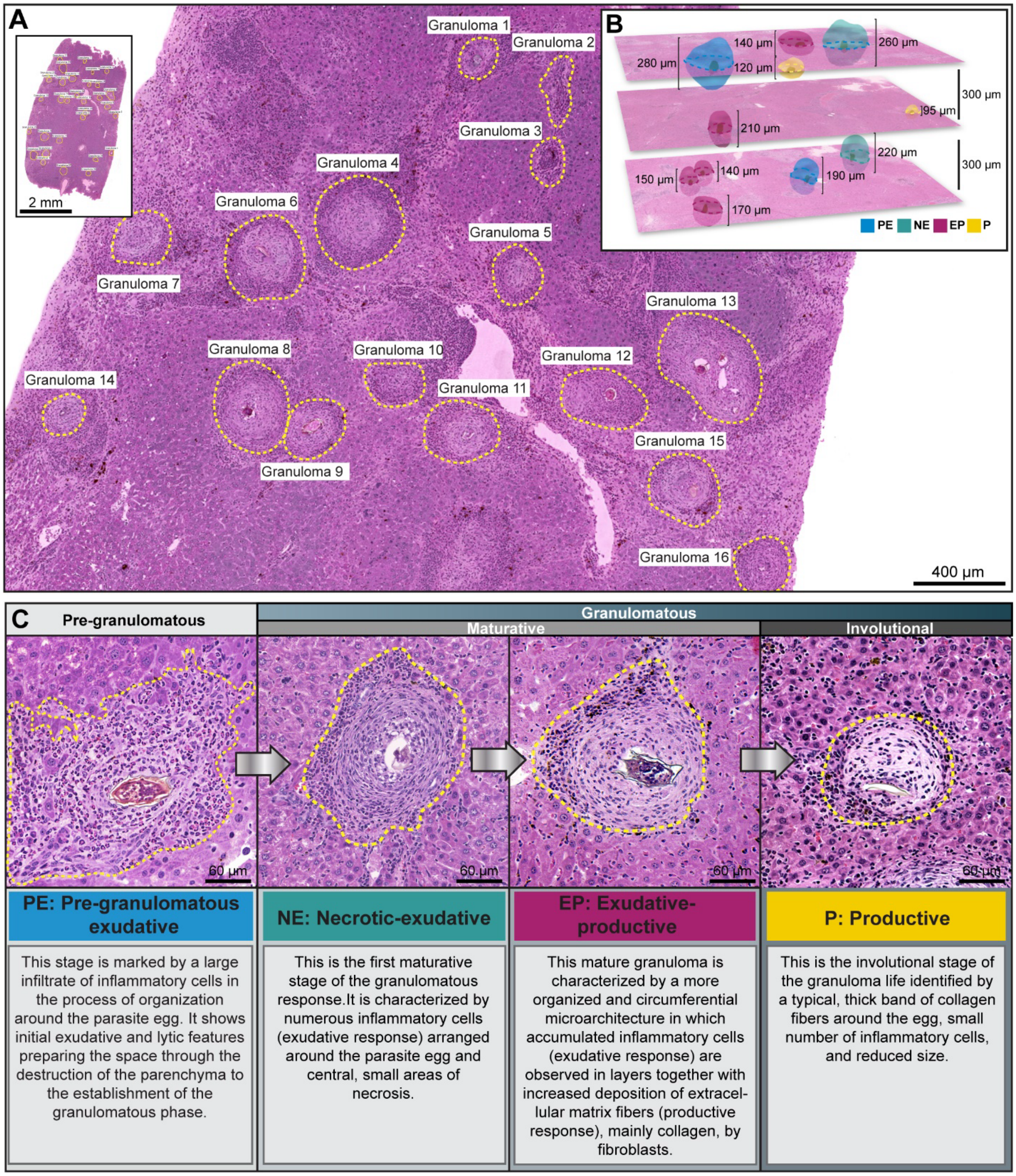

Fig. S1. Distribution and histopathological features of *Schistosoma mansoni* hepatic evolutional granulomas. (**A**) Multiple granulomas (outlined), showing different sizes and at distinct evolutive stages, are observed in a representative H&E-stained section of an infected mouse liver. In (**B**), an illustration depicts, in three-dimensional (3D) view, how these granulomas are distributed in the liver, encompassing three tissue sections with an interval of 300 µm. (**C**) The main characteristics of the pre-granulomatous and granulomatous inflammation are described.

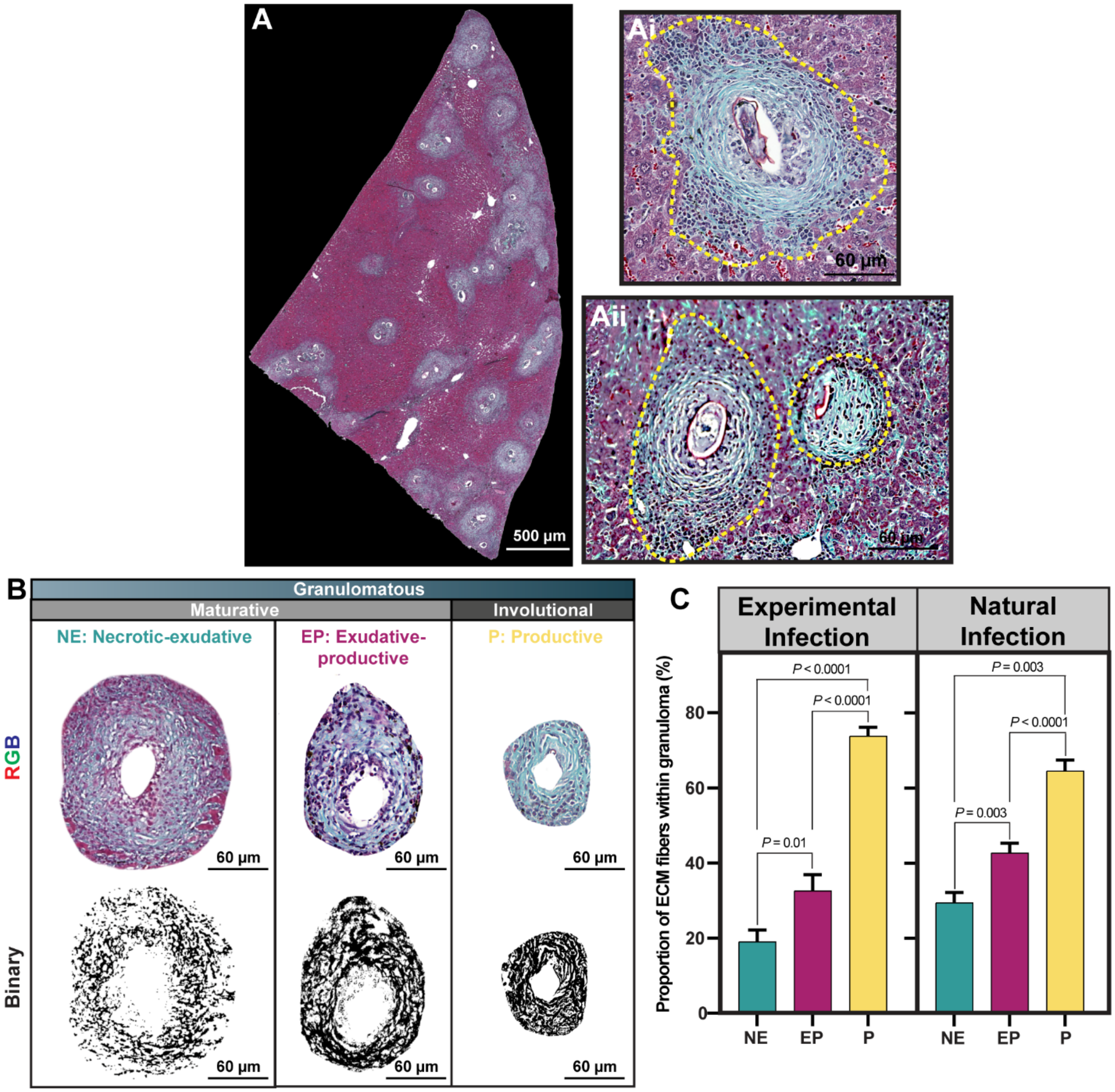

Fig. S2. **Progressive accumulation of extracellular matrix (ECM) during *Schistosoma* granuloma development.** (**A**) Representative WSI scan of the liver after Gomori's trichrome staining. (**Ai** and **Aii**) Higher magnification of the liver in (A) showing representative types of evolving granulomas: Necrotic-Exudative (NE) (Ai), Exudative-Productive (EP) (Aii**,** on the left), and Productive (P) (Aii**,** on the right). Collagen fibers are stained in green. (**B**) Gomori's mask for the automatic calibration of green pixels from Gomori-stained regions to create a binary image. (**C**) Quantitative analysis detected a progressive ECM accumulation during granuloma development in both mouse experimental (acute + chronic) and natural (*Nectomys squamipes*) *Schistosoma mansoni* infections. Results are expressed as means ± SEM. *P* as indicated by Kruskal-Wallis test followed by Dunn's multiple comparisons test. A total of 240 granulomas [120 for the experimental infection (acute + chronic) and 120 for the natural infection; n = 40 per granuloma type (NE, EP, and P)] were analyzed.

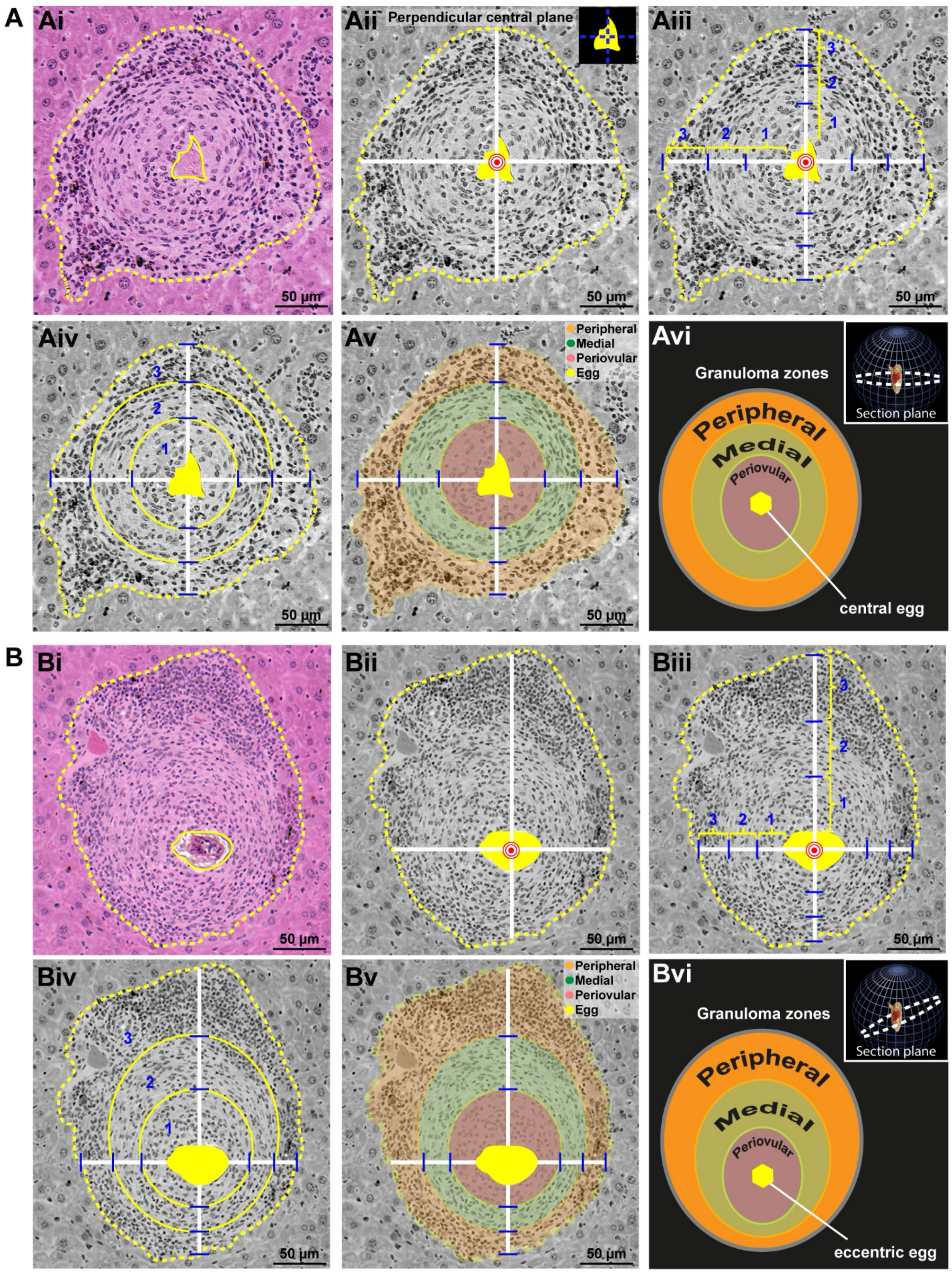

A
Ai
50 µm
Aii
Perpendicular central plane
50 µm
Aiii
3
2
1
50 µm
Aiv
50 µm
Av
Peripheral
Medial
Periovular
Egg
50 µm
Avi
Granuloma zones
Section plane
Peripheral
Medial
Periovular
central egg
B
Bi
50 µm
Bii
50 µm
Biii
50 µm
Biv
50 µm
Bv
Peripheral
Medial
Periovular
Egg
50 µm
Bvi
Granuloma zones
Section plane
Peripheral
Medial
Periovular
eccentric egg

Fig. S3. Digital annotation workflow for granuloma zonation. Sequence showing zonation in representative *Schistosoma* granulomas with a central (**Ai** to **Avi**) or eccentric (**Bi** to **Bvi**) eggs. Granulomas were first outlined (Ai) and (Bi) and two lines were drawn in a perpendicular plane intersecting at the center of the egg, as shown in (Aii and Bii). Note that one line must pass through the major axis of the egg. Then, along these two perpendicular lines, the distance from the eggshell to the granuloma boundary was divided into three equal parts, as indicated by blue marks in (Aiii and Biii). Finally, circles were drawn through each blue mark (division point) (Aiv and Biv). This procedure has established three circular zones: periovular, medial, and peripheral (Av and Bv). Thus, these zones were reliably identified in granulomas with either central (Av and Avi) or eccentric eggs (Bv and Bvi), and the number of eosinophils was quantified within each zone.

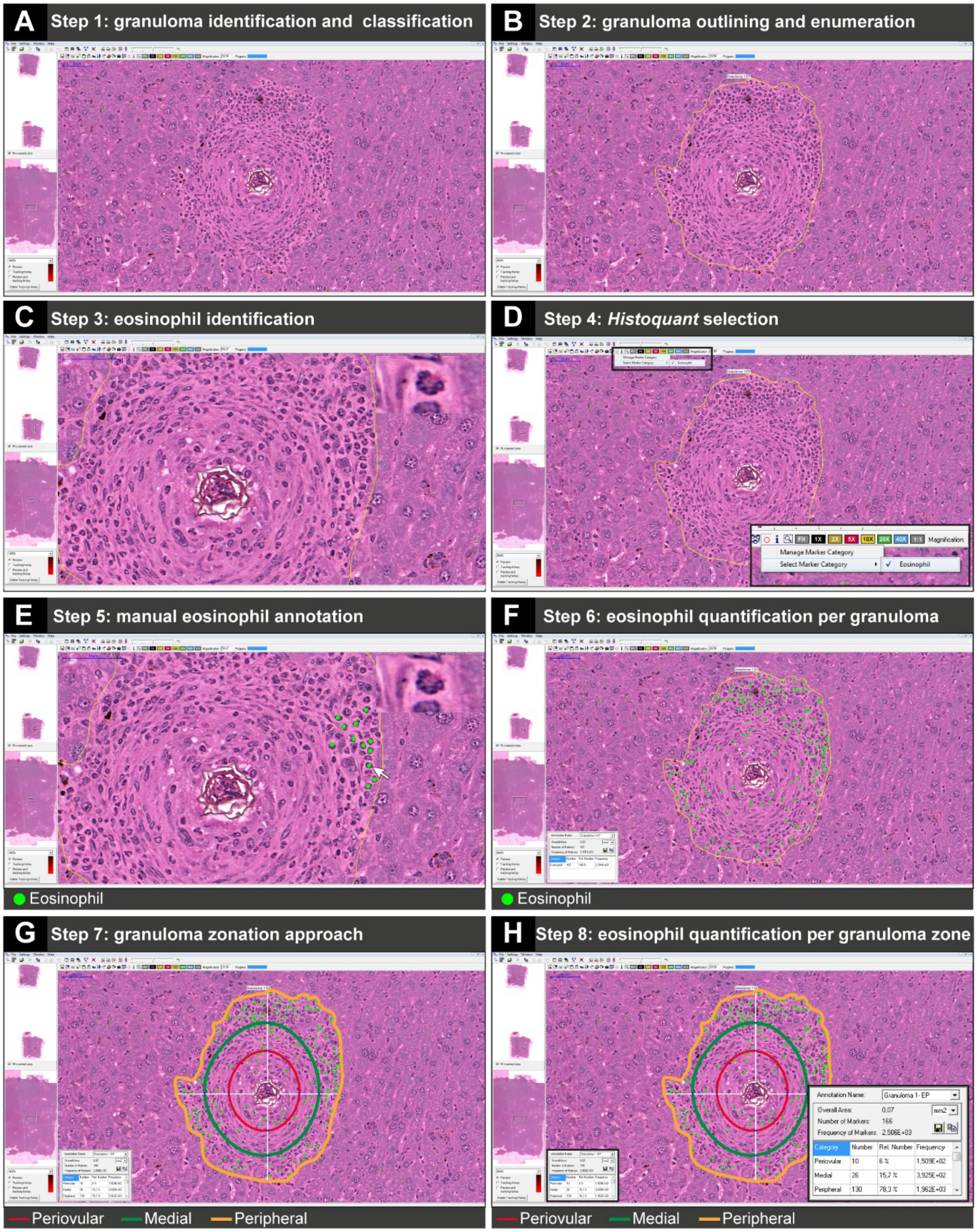
A
Step 1: granuloma identification and classification
B
Step 2: granuloma outlining and enumeration
C
Step 3: eosinophil identification
D
Step 4: Histoquant selection
Fit
1X
2X
5X
10X
20X
40X
1:1
Magnification:
Manage Marker Category
Select Marker Category
Eosinophil
E
Step 5: manual eosinophil annotation
Eosinophil
F
Step 6: eosinophil quantification per granuloma
Eosinophil
G
Step 7: granuloma zonation approach
Periovular
Medial
Peripheral
H
Step 8: eosinophil quantification per granuloma zone
Annotation Name: Granuloma 1 - EP
Overall Area: 0,07 mm2
Number of Markers: 166
Frequency of Markers: 2,506E+03
Category Number Rel. Number Frequency
Periovular 10 6 % 1,509E+02
Medial 26 15,7 % 3,925E+02
Peripheral 130 78,3 % 1,962E+03
Periovular
Medial
Peripheral

Fig. S4. Eosinophil identification and enumeration within hepatic granulomas using Pannoramic Viewer Software. (**A**) In WSI scans of H&E-stained histological sections from the livers of *Schistosoma mansoni*-infected animals, granulomas were identified and classified according to their evolutionary stages as described in Fig. S1. (**B**) Each granuloma was manually outlined using the software's annotation tool and enumerated. (**C**) Eosinophils were identified based on their typical morphological features (segmented nucleus, which stains purple by hematoxylin, and highly acidophilic cytoplasm, which stains pink by eosin). The software displays a panel in the upper-right corner showing at higher magnification the same cell as seen under the cursor, thereby facilitating identification. (**D**) Histoquant (inset), a Pannoramic Viewer's module, was used for eosinophil quantification, and a predefined "Eosinophil" subcategory was selected for analysis. (**E**) Eosinophils were manually annotated by clicking on individual cells and placing a mark on them (green dots). (**F**) By annotating all eosinophils, *Histoquant* automatically has enumerated them. (**G**) The granuloma was then subdivided into three spatial zones (periovular, medial, and peripheral) as described in Fig. S3. (**H**) Eosinophils were enumerated within each zone by *Histoquant*.

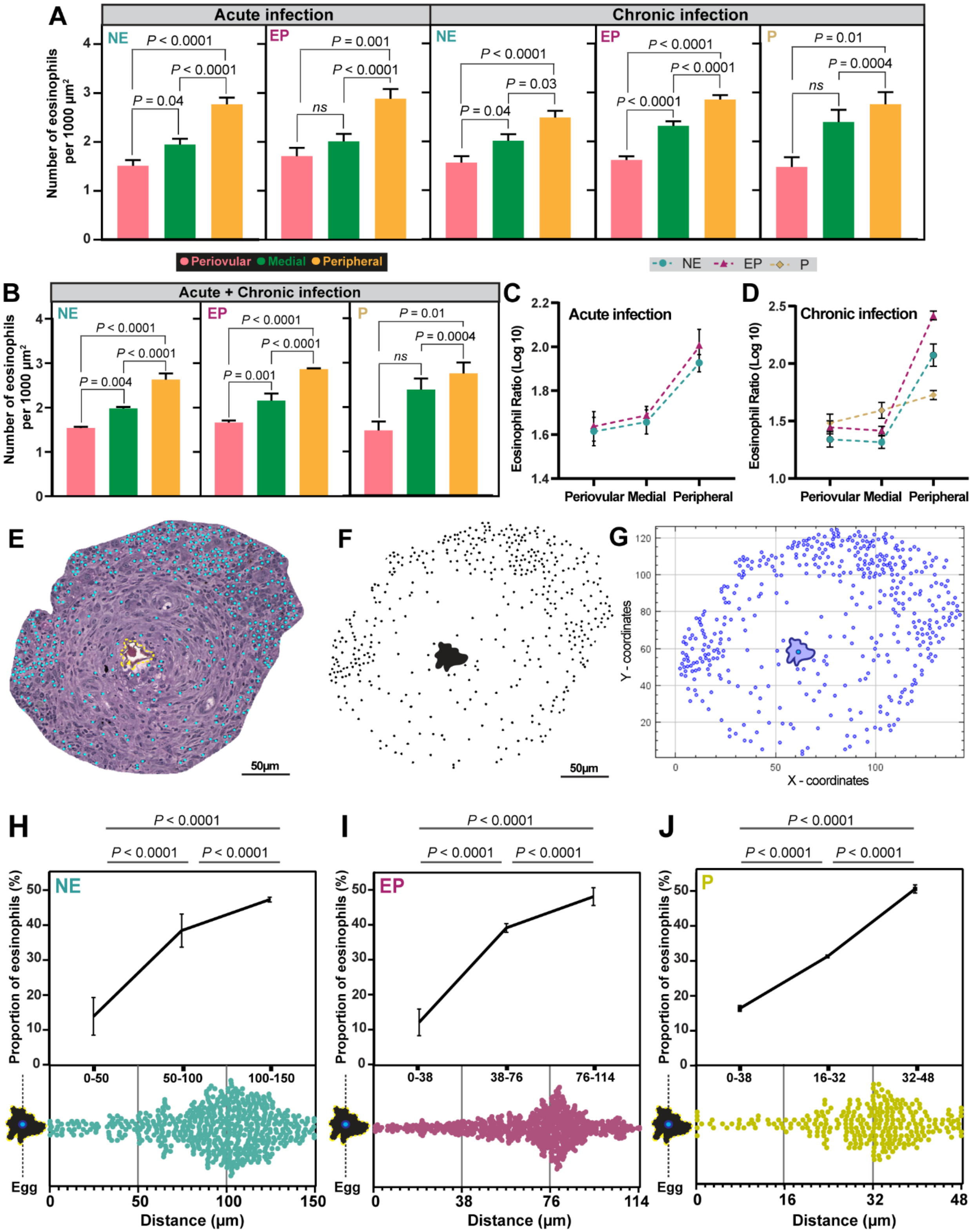

A
Acute infection
Chronic infection
NE
EP
P
Number of eosinophils per 1000 µm²
P < 0.0001
P = 0.04
P = 0.001
ns
P = 0.03
P = 0.01
P = 0.0004
Periovular
Medial
Peripheral
NE EP P
B
Acute + Chronic infection
P = 0.004
C
Acute infection
Eosinophil Ratio (Log 10)
D
Chronic infection
E
F
G
50µm
Y - coordinates
X - coordinates
H
I
J
Proportion of eosinophils (%)
0-50
50-100
100-150
0-38
38-76
76-114
16-32
32-48
Egg
Distance (µm)

Fig. S5. **Quantification and spatial distribution of eosinophils within granulomas from experimental *Schistosoma mansoni* infection in mice.** (**A** and **B**) Number of eosinophils per area (μm^2) of each granuloma zone. WSI analyses using *Histoquant* as described in Fig. S4 showed that eosinophils occupy preferentially the peripheral area of all types of evolutional granulomas (n = 424). (**C** and **D**) The eosinophil ratios calculated for each granuloma zone (n = 1,272 zones from 424 granulomas) showed an increased accumulation of eosinophils in relation to other cells in the peripheral zone. Granuloma zonation was performed as shown in Fig. S3. (**E** to **J**) Distance of individual eosinophils from the egg demonstrating dominance of eosinophils in the granuloma periphery. (E) A representative granuloma with annotated eosinophils (blue dots) is seen in (F) after image segmentation by threshold selection using Fiji software. Each dot corresponds to an individual eosinophil. In (G), spatial coordinates were established from each dot/eosinophil (n = 1,030 eosinophils) and the eosinophil-egg center distances were measured and normalized (0-Maximum). In (H to J), the distance ranges are shown for each type of granuloma: 0-150 µm (NE granulomas, n = 4), 0-114 µm (EP granulomas, n = 5), and 0-48µm (P granulomas, n = 5), in which 0 corresponds to nearest eosinophil distance to the egg. The linear distribution of eosinophils showed that these cells are accumulating in the farthest region from the egg, that is, at the periphery of the granuloma. Results are expressed as means ± SEM. *P* as indicated by one-way ANOVA followed by Tukey's multiple comparisons test. NE, necrotic-exudative; EP, exudative-productive; P, productive.

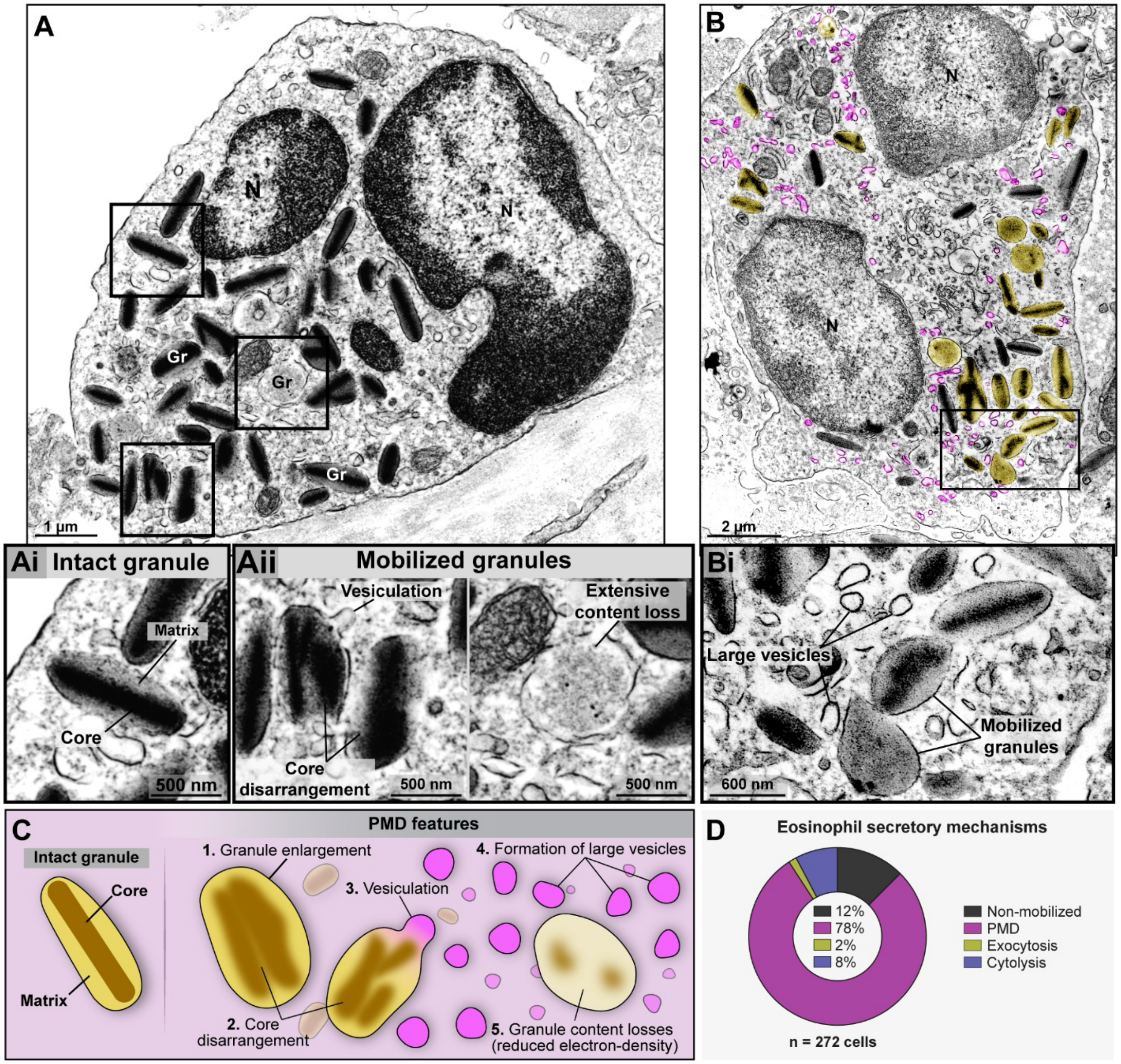

Fig. S6. Eosinophils degranulate through piecemeal degranulation (PMD) within *Schistosoma* granulomas formed in the liver. (**A** and **B**) Ultrastructural features of PMD were consistently found in the cytoplasm of eosinophils spatially localized in the peripheral zone of granulomas. (**Ai**, **Aii** and **Bi**) Higher magnification of the boxed areas in (A) and (B) showing secretory (specific) granules. In (Ai), granules with their intact electron-dense cores are observed. In (Aii and Bi), granules exhibit PMD features, including granule enlargement, core disarrangement, content losses, and formation of large vesicles (80-150 nm). (**C**) Schematic representation comparing an intact granule with granules displaying PMD features. (**D**) Quantitative analyses of the secretory processes shown by eosinophils within the peripheral zone of granulomas. N, nucleus; Gr, secretory granules. A total of 272 eosinophils from both acute and chronic *S. mansoni* infection in mice (n = 6 animals per group) were analyzed.

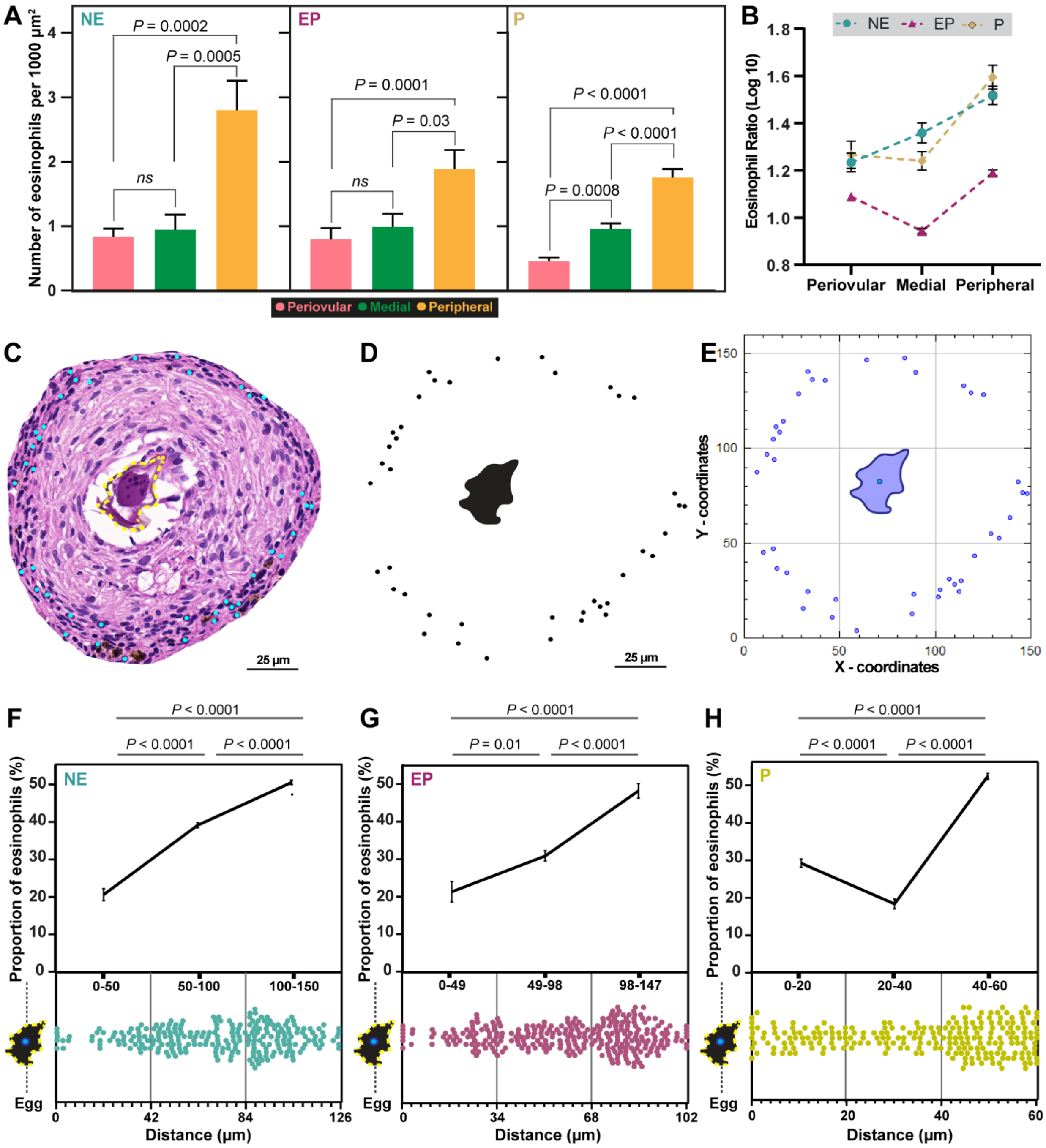

A
NE
EP
P
Number of eosinophils per 1000 µm²
P = 0.0002
P = 0.0005
ns
P = 0.0001
P = 0.03
P < 0.0001
P < 0.0001
P = 0.0008
Periovular
Medial
Peripheral
B
Eosinophil Ratio (Log 10)
Periovular
Medial
Peripheral
C
25 µm
D
25 µm
E
Y - coordinates
X - coordinates
F
G
H
P < 0.0001
P = 0.01
Proportion of eosinophils (%)
0-50
50-100
100-150
0-49
49-98
98-147
0-20
20-40
40-60
Egg
Distance (µm)

Fig. S7. **Quantification and spatial distribution of eosinophils within granulomas from natural *Schistosoma mansoni* infection in *Nectomys squamipes*.** (**A**) Number of eosinophils per area (μm^2) of each granuloma zone. WSI analyses using *Histoquant* as described in Fig. S4 showed that eosinophils occupy preferentially the peripheral area of all types of evolutional granulomas (n = 142). (**B**) The eosinophil ratios calculated for each granuloma zone (n = 426 zones from 142 granulomas) showed an increased accumulation of eosinophils in relation to other cells in the peripheral zone. Granuloma zonation was performed as shown in Fig. S3. (**C** to **H**) Distance of individual eosinophils from the egg demonstrating dominance of eosinophils in the granuloma periphery. (C) A representative granuloma with annotated eosinophils (blue dots) is seen in (**D**) after image segmentation by threshold selection using *Fiji* software. Each dot corresponds to an individual eosinophil. In (E), spatial coordinates were established from each dot/eosinophil (n = 750 eosinophils) and the eosinophil-egg center distances were measured and normalized (0-maximum). In (F to H), the distance ranges are shown for each type of granuloma: 0-126 μm (NE granulomas, n = 6), 0-102 μm (EP granulomas, n = 5), and 0-60 μm (P granulomas, n = 5), in which 0 corresponds to nearest eosinophil distance to the egg. The linear distribution of eosinophils showed that these cells are accumulating in the farthest region from the egg, that is, at the periphery of the granuloma. Results are expressed as means ± SEM. *P* as indicated by one-way ANOVA followed by Tukey's multiple comparisons test. NE, necrotic-exudative; EP, exudative-productive; P, productive.

Table S1. Histopathological data from mice with acute and chronic *Schistosoma mansoni* infection.

	Acute infection (n = 4 animals)			Chronic infection (n = 4 animals)				Acute + Chronic infection (n = 8 animals)			
**Granuloma Stage**	PE	NE	EP	PE	NE	EP	P	PE	NE	EP	P
**Mean number of granulomas per tissue section (Mean ± SEM)**	0.67 ± 0.28	7.67 ± 0.65	4.25 ± 0.60	0.17 ± 0.17	4.17 ± 0.85	16.00 ± 1.38	3.92 ± 0.85	0.41 ± 0.17	5.92 ± 0.64	10.12 ± 1.43	3.92 ± 0.85
**Total number of granulomas**	8	92	51	2	50	192	47	10	142	243	47
	151			291				442			
**Mean granuloma area (Mean ± SEM; mm^2)**	0.089 ± 0.001	0.081 ± 0.004	0.060 ± 0.004	0.059 ± 0.006	0.049 ± 0.003	0.033 ± 0.001	0.018 ± 0.001	0.051 ± 0.001	0.067 ± 0.002	0.038 ± 0.001	0.018 ± 0.001
**Total granuloma area (mm^2)**	0.27	7.16	2.71	0.04	2.45	6.64	0.85	0.31	10.30	10.18	0.85
**Proportion of tissue occupied by granulomas (%)**	0.2	3.81	2.61	0.19	1.20	3.19	0.41	0.09	3.03	2.98	0.41
	6.62			4.99				6.35			
**Mean number of granulomas/ tissue area (Mean ± SEM; n/mm^2)**	0.06 ± 0.02	0.72 ± 0.09	0.38 ± 0.05	0.01 ± 0.01	0.25 ± 0.05	0.94 ± 0.08	0.24 ± 0.06	0.03 ± 0.01	0.49 ± 0.07	0.66 ± 0.08	0.24 ± 0.06
**Mean number of cells/granuloma (Mean ± SEM)**	192.90 ± 33.75	267.30 ± 11.98	214.00 ± 14.30	118.00 ± 26.32	188.20 ± 13.09	140.50 ± 7.18	64.13 ± 4.63	177.90 ± 28.08	239.10 ± 9.42	135.10 ± 4.09	64.13 ± 4.63
**Total number of cells**	3,086	48,118	20,760	472	18,816	44,966	6,028	3,558	66,934	65,726	6,028
	71,964			70,282				142,246			
**Mean number of eosinophils/ granuloma (Mean ± SEM)**	200.40 ± 49.00	164.10 ± 10.33	144.38 ± 13.53	130.50 ± 58.50	100.60 ± 8.26	74.08 ± 2.92	35.96 ± 3.64	186.40 ± 40.71	141.60 ± 7.71	88.92 ± 4.08	35.96 ± 3.64
**Total number of eosinophils**	1,603	14,933	7,384	261	5,030	14,224	1,690	1,864	19,963	21,608	1,690
	23,920			21,205				45,125			
**Mean liver section area (Mean ± SEM; mm^2)**	14 ± 0.81			11 ± 0.67				17 ± 0.82			
**Total liver section area (mm^2)**	540			828				1,368			
**Total number of eggs/ tissue area (n/ mm^2)**	2.81			1.88				2.35			
**Total number of eggs**	334			381				715			

Table S2. Histopathological data from *Nectomys squamipes* naturally infected with *Schistosoma mansoni*.

	N. squamipes (n = 4)			
**Granuloma Stage**	PE	NE	EP	P
**Mean number of granulomas per tissue section (Mean ± SEM)**	1.89 ± 0.59	1.44 ± 0.29	0.89 ± 0.31	11.56 ± 1.68
**Total number of granulomas**	17	13	8	104
	142			
**Mean granuloma area (Mean ± SEM; mm²)**	0.031 ± 0.005	0.040 ± 0.005	0.023 ± 0.004	0.016 ± 0.001
**Total granuloma area (mm²)**	0.38	0.51	0.19	1.66
**Proportion of tissue occupied by granuloma (%)**	0.33	0.45	0.16	1.44
	2.38			
**Mean number of granulomas/ tissue area (Mean ± SEM; n/ mm²)**	0.015 ± 0.05	0.12 ± 0.03	0.07 ± 0.03	0.90 ± 0.13
**Mean number of cells/granuloma (Mean ± SEM)**	121.9 ± 16.61	136.2 ± 21.18	118.6 ± 28.90	31.64 ± 2.23
**Total number of cells**	4,145	3,405	1,897	5,948
	15,395			
**Mean number of eosinophils/ granuloma (Mean ± SEM)**	71.41 ± 13.27	60.85 ± 6.91	39.63 ± 16.83	10.38 ± 1.04
**Total number of eosinophils**	1,214	791	317	1,080
	3,402			
**Mean liver section area (Mean ± SEM; mm²)**	14.01 ± 0.52			
**Total liver section area (mm²)**	1,033			
**Total number of eggs/ tissue area (n/ mm²)**	1.49			
**Total number of eggs**	170			

Table S3: Quantitative data from 3D reconstructed granulomas.

Infection model	Granuloma Stage	Total number of sections	Granuloma thickness (µm)	Granuloma volume (10^{-3} mm^3)	Total number of eosinophils	Eosinophils/ granuloma volume (eos/10^{-3} mm^3)
**Swiss mice**	**PE**	60	180	35.9	6,243	173.9
	**NE**	69	207	99.4	5,689	57.2
	**EP**	67	201	50.6	3,265	64.5
	**P**	48	144	27.1	462	17.0
Nectomys Squamipes	**PE**	89	267	108.2	16,570	153.1
	**NE**	74	222	163.8	3,022	18.4
	**EP**	81	243	80.3	5,303	66.0
	**P**	44	132	16.4	251	15.3

Movie S1. Three-dimensional (3D) reconstruction and rendering of an entire hepatic granuloma from a mouse infected with *Schistosoma mansoni.* A representative granuloma (exudative-productive/EP) from a WSI scan of the liver tissue (H&E-stained) was serially sectioned (n = 67 sections), reconstructed and modeled in 3D. The granuloma was digitally segmented from the liver tissue to visualize the spatial organization of eosinophils within granuloma. The *Schistosoma* egg and eosinophils were marked in yellow and red, respectively.

Movie S2. Three-dimensional (3D) reconstructions and rendering of entire representative evolutional granulomas of a mouse infected with *Schistosoma mansoni.* Representative pregranulomatous-exudative (PE, n = 60 sections), necrotic-exudative (NE, n = 69 sections), exudative-productive (EP, n = 67 sections), and productive (P, n = 48 sections) granulomas from WSI scans of the liver tissue (H&E-stained) were serially sectioned, reconstructed and modeled in 3D. The *Schistosoma* egg and eosinophils were marked in yellow and red, respectively.

Movie S3. Three-dimensional (3D) reconstructions and rendering of entire representative hepatic evolutional granulomas of a *Nectomys squamipes* (water rat) naturally infected with *Schistosoma mansoni.* Representative pregranulomatous-exudative (PE, n = 89 sections), necrotic-exudative (NE, n = 74 sections), exudative-productive (EP, n = 81 sections), and productive (P, n = 44 sections) granulomas from WSI scans of the liver tissue (H&E-stained) were serially sectioned, reconstructed, and modeled in 3D. The *Schistosoma* egg and eosinophils were marked in yellow and red, respectively.
